# Fibrotic Changes in Rhegmatogenous Retinal Detachment

**DOI:** 10.3390/ijms26031025

**Published:** 2025-01-25

**Authors:** Niina Harju, Anu Kauppinen, Sirpa Loukovaara

**Affiliations:** 1 School of Pharmacy, Faculty of Health Sciences, University of Eastern Finland, 70210 Kuopio, Finland; anu.kauppinen@uef.fi; 2 Unit of Vitreoretinal Surgery, Department of Ophthalmology, Helsinki University Central Hospital, 00029 Helsinki, Finland; 3 Individualized Drug Therapy Research Program, University of Helsinki, 00014 Helsinki, Finland

**Keywords:** rhegmatogenous retinal detachment, proliferative vitreoretinopathy, epithelial–mesenchymal transition, fibrosis, epiretinal membrane

## Abstract

Rhegmatogenous retinal detachment (RRD) is a sight-threatening condition involving retinal detachment and the accumulation of fluid in the subretinal space. Proliferative vitreoretinopathy (PVR) is a pathologic complication that develops after RRD surgery, and approximately 5–10% of RRD cases develop post-operative PVR. Prolonged inflammation in the wound healing process, epithelial–mesenchymal transition (EMT), retinal pigment epithelial (RPE) cell migration and proliferation, and epiretinal, intraretinal, and subretinal fibrosis are typical in the formation of PVR. RPE cells undergo EMT and become fibroblast-like cells that migrate to the retina and vitreous, promoting PVR formation. Fibroblasts transform into myofibroblasts, which promote fibrosis by overproducing the extracellular matrix (ECM). RPE cells, fibroblasts, glial cells, macrophages, T lymphocytes, and increased ECM production form contractile epiretinal membranes. Cytokine release, complement activation, RPE cells, glial cells, and endothelial cells are all involved in retinal immune responses. Normally, wounds heal within 4 to 6 weeks, including hemostasis, inflammation, proliferation, and remodeling phases. Properly initiated inflammation, complement activation, and the function of neutrophils and glial cells heal the wound in the first stage. In a retinal wound, glial cells proliferate and fill the injured area. Gliosis tries to protect the neurons and prevent damage, but it becomes harmful when it causes scarring. If healing is complicated, prolonged inflammation leads to pathological fibrosis. Currently, there is no preventive treatment for the formation of PVR, and it is worth studying in the future.

## 1. Introduction

Rhegmatogenous retinal detachment (RRD) is a vision-threatening condition, especially when associated with proliferative vitreoretinopathy (PVR) [1]. Symptoms of RRD include black spots, floaters, flashes of light, and ultimately vision loss [2,3,4]. It can develop from various ocular conditions (e.g., high myopia), after ocular surgery (e.g., cataract, glaucoma, refractive surgery), be associated with a genetic background, or be caused by ocular trauma [2,3,5,6,7,8,9,10,11,12]. PVR is a pathological complication that develops after RRD and its surgery by the formation of proliferative scar membranes in the posterior vitreous or epi-, intra, and/or subretinally [13,14,15,16]. The formation of PVR is a complex and multifactorial process influenced by various cells and intra- and extraocular factors [16]. Long-standing and extensive retinal detachment, vitreous hemorrhage, poor initial vision (e.g., macula off), cataract, vitreous traction, high aqueous and vitreous protein content, and the pre-operative inflammatory phase increase the risk of developing PVR after RRD surgery [17,18,19,20,21]. The acute response to retinal detachment is the proliferation of non-neuronal retinal cells (e.g., astrocytes, Müller cells, and microglia) [14,22,23]. Typical for the development of PVR is inflammation during the wound healing process, epithelial–mesenchymal transition (EMT), the migration and proliferation of retinal pigment epithelial (RPE) cells, and fibrotic formation, i.e., scarring [24,25]. The more advanced form of PVR formation associated with RRD is characterized by platelet-mediated wound healing, increased apoptosis, and the rearrangement of cell adhesion molecules, among others [26].

Retinal detachment can be divided into rhegmatogenous, tractional, and exudative types [27]. In each of them, the neuroretina separates from the RPE and choroid layer, leading to the rapid degeneration of photoreceptor outer segments [27,28,29]. A retinal break, tear, or hole is a typical finding during RRD [2,6,8,30]. In contrast, in tractional and/or combined rhegmatogenous–tractional retinal detachment, contractile epiretinal membranes are formed in the vitreous or the surface of the retina, which induces detachment of the neuroretina from the RPE cell layer [31,32,33]. In exudative retinal detachment, fluid accumulates under the retina between the neuroretina and RPE layers [34,35,36]. The worldwide incidence of RRD is increasing and is approximately 12.2 cases per 100,000 (1 per 10,000) [37,38]. About 5–10% of RRD cases develop post-operative PVR, suggesting an irregular scarring process [18,19,39,40]. Some studies have reported even higher (e.g., 26.9%, 29.4%, and 52.9%) incidences of PVR after RRD surgery, and factors such as age, pre-operative ocular condition, and ethnicity may influence the likelihood of developing post-operative PVR [17,41]. Although PVR can occur pre-operatively, it is usually much more common post-operatively as a side effect of eye surgery [16]. The pre-operative form affects the prognosis for the development of a severe form after surgery [16,42,43].

**Figure 1:**
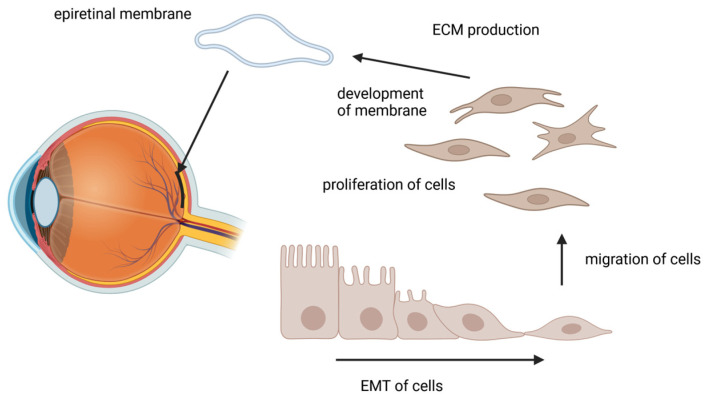
Schematic picture illustrating the formation of the epiretinal membrane in the macular area (Created in BioRender. Harju, N. (2025) https://BioRender.com/f95o003; access from 22 January 2025).

PVR is classified into grades A to D as follows: minimal (A), moderate (B), marked (C), and massive (D) [13,42]. Clinical findings in grade A include vitreous mist and pigment cells/clusters, and, in B, retinal wrinkles, rolling edges of retinal tears, and retinal vascular stiffness [44]. In grade C, full-thickness retinal folds appear in one to three quadrants, and, typically, in grade D, retinal folds appear in all four quadrants [13,44]. In grade D, the folding occurs concurrently with retinal thickening and is poorly repaired by surgery [16]. Grades C and D are classified as severe forms of PVR [18,19,42]. The formation of PVR proceeds roughly in the following order: 1. EMT and the migration of cells (e.g., RPE and glia cells) to the vitreous and retinal surface, 2. the proliferation of migrated cells, 3. the development of membranes containing cells, 4. the contraction of the membrane, 5. the production of collagen as an extracellular matrix (ECM) material in the membranes, and 6. the formation of rigid and fixed folds in the membranes on the retina and protrusion into the vitreous (Figure 1) [13,45]. Currently, no effective preventive or therapeutic strategies are available to reduce the risk of developing PVR in RRD eyes.

## 2. From RRD to PVR Formation

In RRD, vitreous fluid typically accumulates subretinally between the RPE cells and photoreceptors [2]. As PVR develops, neuroretina tightening and epi- or preretinal, intraretinal, and/or subretinal fibrosis lead to retinal wrinkling and folding [16,26]. Cytokines associated with inflammation are typical findings in the subretinal space of retinal detachment eyes and the vitreous fluid of PVR eyes [46,47]. After retinal detachment repair surgery, many remodeling changes attempt to balance the retinal state, including rod photoreceptors, ganglion cells, and horizontal cells growing neurites into the subretinal space and into the vitreous for targeted or non-targeted synaptic interactions [29]. Müller cells proliferate and reorganize in the subretinal space during retinal detachment or in the vitreous after reattachment [29].

Understanding the processes involved in cell proliferation, tissue remodeling, and scar formation in RRD is of great importance. In the most severe form of PVR-RRD, contractile membranes form in the vitreous and on both sides of the retina, inducing retinal traction and causing re-detachment [16,24]. In vitreous samples from RRD eyes with PVR, the mRNA levels of Fas (CD95) and the tumor necrosis factor (TNF)-related apoptosis-inducing ligand (TRAIL) and the protein levels of transforming growth factor-beta2 (TGF-β2) differ significantly from those of macular hole patients, indicating that Fas, TRAIL, and TGF-β2 are early mediators of PVR formation [48]. Dieudonné et al. showed that subretinal TGF-β2 levels are increased in retinal detachment but decreased after postoperative PVR [49]. Immunoreactive endothelin one (IR-ET-1) is also increased in the vitreous of RRD eyes with PVR [50]. Increased levels of vimentin and heme oxygenase 1 have been observed in the vitreous of RRD eyes with PVR formation [51].

Collagen and its related proteins (TGFβ-1, matrix metalloproteinase [MMP] inhibitor 1, collagen alpha-1) are increased in the vitreous of RRD patients [26]. Öhman et al. studied changes in the vitreous protein profile when comparing acute RRD with chronic (RRD with PVR) disease [26]. Of the 1177 proteins measured, 313 were increased in RRD eyes, and 29 proteins were differentially expressed in the vitreous of PVR eyes, 15 of which were upregulated (Table 1) [26]. Öhman et al. showed that RRD patients have unique vitreous protein profiles related, e.g., to apoptosis, rhodopsin, glycolysis, and wound healing [26]. They found that proteins involved in both activating and deactivating phototransduction pathways and visual perception were increased in the RRD vitreous samples [26]. Tissue inhibitors of metalloproteinases (TIMPs) are also increased in the retina after retinal detachment [52].

Retinal detachment-related PVR formation can be defined in different phases: ischemic and inflammatory phase, apoptotic phase, cell transformation, migration and proliferation phase, and scar contraction phase [53]. The ischemic phase involves blood–retinal barrier (BRB)-related blood flow disturbances that rapidly lead to photoreceptor death [22,53,54]. Retinal capillary leaking can persist for several months after retinal detachment (RD) repair [55]. RPE and Müller cells respond rapidly after RD, e.g., by activating the expression of extracellular signal-regulated kinases (ERKs) and activating protein-1 (AP-1) when cAMP response element-binding protein (CREB) is activated in photoreceptors [56]. Basic fibroblast growth factor 2 (FGF-2, bFGF-2), the signal transducer and activator of transcription (STAT), and nuclear factor-κB (NF-κB) are activated in the retina immediately after RD [56]. Within 3 days of retinal detachment, the photoreceptor number and photoreceptor outer segment length were shown to decrease due to apoptosis, i.e., via intrinsic apoptotic pathways [22]. Cell proliferation is also observed 3 days after retinal detachment [22]. Typically, the cells that proliferate after retinal detachment are non-neuronal cells, e.g., RPE cells, microglia, endothelial cells, Müller cells, and astrocytes [22]. Retinal reattachment reduces the Müller cell proliferation, but RPE cell proliferation may continue [22]. During the inflammatory phase, serum-related factors (e.g., thrombin) are released, and immune cells (e.g., macrophages) infiltrate the vitreous [53,57,58]. Thrombin has been shown to induce the release of interleukin (IL)-6, IL-8, monocyte chemoattractant protein (MCP)-1, granulocyte-macrophage colony-stimulating factor (GM-CSF), and platelet-derived growth factor (PDGF)-BB from RPE cells [57]. After retinal detachment, photoreceptors die via intrinsic apoptotic pathways [59]. For example, pro-apoptotic Fas is increased in the vitreous during the formation of PVR associated with retinal detachment [48,53]. TGF-β levels are also elevated either as a pro-apoptotic or as a cell survival and proliferation factor [60]. Mitotically inactive RPE cell proliferation is thought to begin rapidly after retinal detachment [61]. The migration and proliferation phase of cells include e.g., the TGF-β-induced EMT of RPE cells to produce fibroblast-like cells that migrate to the retina or the vitreous [53]. Fibroblasts can originate from RPE cells that have undergone EMT, Müller cells via the glial–mesenchymal transition, or from the bloodstream [53,62,63,64]. Cells (e.g., fibroblasts, RPE cells, Müller cells, T lymphocytes, macrophages, astrocytes, and microglia) collectively form epiretinal membranes with increased ECM production [53]. The main components of the ECM are collagens, fibronectins, glycosaminoglycans, and proteoglycans [65]. The EMT of cells and ECM accumulation is an important factor in the development of PVR [25].

### 2.1. EMT of Cells

EMT is a normal process in embryogenesis (EMT1) and wound healing but is also associated with fibrosis (EMT2) and cancer progression and metastasis (EMT3) [66]. Cellular EMT involves the loss of cell polarity associated with the epithelial cell phenotype and changes in ECM production [65]. The GTPases Rho, Rac, and Cdc42 mediate the change from apical–basal polarity to front–rear polarity [67]. The polarized structure of RPE cells is maintained by the adhesion proteins E-cadherin, occludin, and claudin [39]. EMT results in the conversion of membrane-associated E-cadherin to N-cadherin and the cytoskeleton protein cytokeratin to vimentin and α-smooth muscle actin (α-SMA) [68]. RPE cells undergo EMT, becoming fibroblast-like as tight junctions between cells are weakened [64]. Mesenchymal cells lack tight junctions and have a phenotype that is altered to front–rear polarity, allowing cell migration [67,69]. Properly formed epithelial cells provide a barrier to tissues, have a cuboidal and apical-basal morphology, and organize tight structures [68]. Mesenchymal cells are able to transfer back into epithelial cells to form a secondary epithelium [69].

Fibroblast-like cells differentiate into myofibroblasts under the influence of TGF-β and Rho signals [64]. Both fibroblasts and myofibroblasts can be contractile cells in epiretinal membranes [64]. TGF-β is a major known EMT activator and acts by activating the suppressor of mothers against the decapentaplegic (Smad) complex, which co-operates with transcription factors (e.g., snail family proteins snail1 [Snail], twist-related protein 1 [Twist], zinc finger E-box binding [Zeb], and forkhead box [FOX]) [67,68]. RPE-derived cells migrate, proliferate, and modify the ECM and are part of contractile epiretinal membranes [64]. In EMT, transcription factors (e.g., Snail, Snail2 [Slug], Twist, and Zeb) downregulate epithelial markers (E-cadherin, claudin, occludin, mucin-1, phosphatase and tensin homolog [PTEN], and Raf kinase inhibitory protein [RKIP]) and induce mesenchymal markers (N-cadherin, vimentin, vitronectin, matrix metalloproteases, and beta 1 and 3 integrins) [39,70]. For example, TGF-β-induces EMT by downregulating E-cadherin and zona occluding (ZO-1) in RPE cells concomitant with increased expressions of Snail, fibronectin, and α-SMA [71,72]. The disruption of tight junctions leads to activation of the Hippo and β-catenin/wingless-type MMTV integration site family (Wnt) signaling pathways and the transcription factors Zeb 1, Snail, and ZO-1-associated Y-box factor (ZONAB), which induce the EMT of RPE cells [64]. Myofibroblasts associated with scar tissue have elevated levels of α-SMA, a marker of collagen production [73,74]. Increased levels of Snail are also found in the epiretinal membranes of PVR [71]. N-cadherin levels are increased in RPE cells, photoreceptors, and the external limiting membrane already after retinal detachment and decrease when the retina reattaches [75]. The EMT of cells induces invasive and migratory markers, in which cell–cell interactions and interactions with the ECM are reduced [39,76].

In epiretinal membranes from patients with PVR, TGF-β levels are highly abundant, causing EMT and ECM production mediated by the phosphatidylinositol 3′-kinase (PI3K)/protein kinase B (Akt) and jagged canonical Notch ligand 1 (Jagged)/Notch pathways [39,77,78,79]. The jagged/Notch pathway regulates the TGF-β mediated EMT of RPE cells through Snail, Slug, and Zeb1 [79]. TGF-β induces collagen 1-mediated ECM remodeling, more specifically fibrogenesis [72]. TGF-β also activates the ERK1/2 pathway and induces levels of phosphorylated p-38 mitogen-activated protein kinases (MAPKs) and heat shock protein 27 (hsp27) in RPE cells [72,80]. p38 MAPK is involved in many cellular processes, including inflammation, cell differentiation, cell death, cell cycle, and senescence [81]. For example, Müller cells produce TGF-β in the retina [82]. TGF-β promotes PVR formation by inducing the mitogenic effects of other growth factors, mediating contraction, increasing ECM production, and inducing fibrosis but also has features of hyalocyte–gel contraction [78,83]. PDGF promotes the formation of epiretinal membranes and has properties as a mitogenic and chemotactic agent, while FGF induces cell proliferation [83]. Micro RNAs (miRNAs; e.g., miR-29b and miR-124) promote PVR by regulating the EMT and cell migration of RPE cells, e.g., miR-29b correlates positively with E-cadherin levels and negatively with α-SMA [84,85,86]. During RRD, miR-21 and miR-34 are positively correlated with the duration of symptoms in retinal detachment, while miR-146a is increased in RRD with PVR [87]. The expressions of miR-143-3p, miR-224-5p, miR-361-5p, miR-452-5p, miR-486-3p, and miR-891a-5p have been shown to correlate with the worsening of PVR [88].

### 2.2. Formation of Epiretinal Membranes

Scarring accompanied by ischemia, inflammation, and cell proliferation, leads to epiretinal membrane formation and gliosis, causing retinal traction and wrinkling [39]. The membranes that contain dense collagen and embedded cells ultimately lead to distortion of the internal limiting membrane (ILM) [39]. Cytokines and growth factors, e.g., promote the EMT of RPE cells, which migrate through retinal breaks into the vitreous or retinal surface, leading to the formation of contractile membranes [39]. Matricellular proteins may play a crucial role in RPE cell detachment from the Bruch’s membrane [89]. The detached retina causes neuroretinal cell death, leading to the formation of glial scars (gliosis, including Müller cell hypertrophy) [39].

#### 2.2.1. Cells in Epiretinal Membranes

Within a few days, retinal detachment induces rapid cell proliferation (e.g., RPE cells, astrocytes, Müller cells, pericytes, retinal vascular endothelial cells, and macrophages), which is an early event in forming PVR-related membranes [61,90,91]. The resulting membranes or detached retina contain cells that are positive for cytokeratin, suggesting that they incorporate RPE cells that have undergone EMT [39,75]. Cytokeratin is the predominant epithelial cytoskeletal protein that supports cell–cell interactions, whereas vimentin is predominant in mesenchymal cells and favors cell migration [66]. PVR membranes associated with RRD include RPE cells, melanocytes, myofibroblasts, macrophages, Müller cells, activated microglia, astrocytes, T lymphocytes, myeloid cells, hyalocytes, preadipocytes, Myo/Nog cells, and progenitor and stem cells (Table 2) [39,47,58,89,92,93,94,95,96,97]. Neutrophils, B lymphocytes, immunoglobulins, and complement factors have not been found in PVR membranes [98]. Subretinal or epiretinal membranes contain T lymphocytes but not B lymphocytes, neutrophils, or complement components [95,98]. The membranes formed are dynamic complexes in which the cellular and matrix components are constantly changing, and the cells contained in the membrane also vary between individuals [93,99]. In membranes, apoptotic markers have also been detected, suggesting a contribution to the development of PVR membranes [48].

Myofibroblasts and RPE cells are proposed to be the major cell types of the formed membranes [100,101]. For example, epithelial cells have been proposed to transdifferentiate through EMT into myofibroblasts and migrate into the formed membranes [74]. RPE-derived fibroblasts are also proposed to transform into myofibroblast-like cells [64]. Both fibroblasts and myofibroblasts are important determinants of epiretinal PVR membranes [64]. Myofibroblasts are suggested to transdifferentiate from local fibroblasts, epithelial cells, endothelial cells, hyalocytes, glia cells, Myo/Nog cells, myeloid cells, or pericytes, and they are typically present in all contractile epiretinal membranes [73,74,92,96,97,102]. Early in membrane formation, fibroblasts, RPE cells, and glial cells are present, but, when the disease progresses, fibroblasts and cells with markers of degeneration increase, and RPE cells decrease [99]. M2 macrophages, which have anti-inflammatory and tissue remodeling properties, are also found in PVR membranes [58]. Nevertheless, activated macrophages and microglia have been suggested to exacerbate retinal detachment [103]. Subretinal fluid tends to induce the macrophage-mediated phagocytosis of dying RPE cells [47]. Macrophages have also been shown to differentiate into fibroblast-like cells during PVR formation [104].

Zhao et al. suggested that hyalocytes and Müller cells are the main contributors to the proliferation of epiretinal-associated cells in the ILM of RRD-PVR patients, but they also observed other glial cells and fibroblasts [102]. The ILM between the vitreous and the retina is an optimal surface for the formation of epiretinal membranes, but it also mediates vitreoretinal traction in scar formation [102]. Hyalocytes are normal macrophage-like cells located at the back of the vitreous near the ILM and contribute to cell proliferation and migration in the formation of the PVR membrane and subsequent retinal shrinkage [105,106].

#### 2.2.2. Molecular Markers of Epiretinal Membranes

Many different growth factors and cytokines influence epiretinal membrane formation (Table 3) [101]. In epiretinal proteomic analysis, ECM and structural organization pathways are common, and, for example, fibronectin is frequently upregulated and expressed with little variation between membranes [107,108]. Ioachim et al. detected tenascin, fibronectin, laminin, and collagen IV in the ECM of PVR membranes [109]. Epiretinal membranes also contain metalloproteinases and growth factors (e.g., scatter factor/hepatocyte growth factor family) [89]. Migrated RPE cells in epiretinal membranes are detected, e.g., by cytokeratin-18 (i.e., positive for RGE53), a marker of migratory cells, but there are also RGE53-negative RPE cells [94]. RPE migration into epiretinal membranes is regulated, at least in part, by thrombospondin 1 (TSP1) and SPARC, which reduce RPE cell adhesion to the matrix, allowing cell migration and morphological changes [89,94]. RPE cells also produce extracellular collagen and fibronectin [101]. Activated, fibroblast-derived myofibroblasts express α-SMA and are strongly associated with contractile scars [63,100]. Myofibroblasts also express CD44 in their membranes, Snail and hydrogen peroxide-inducible clone-5 (Hic-5) in the nucleus, fibroblast activation protein (FAP), interleukin-13 receptor α2 (IL-13Rα2), and the receptor for advanced glycation end products (RAGE) in the cytoplasm [100]. Fibroblasts can transform to myofibroblasts and co-express α-SMA and the fibroblast-associated markers CD34, CD45, CCR7, CXCR4, CCL21, and CXCL12 [63]. The ILM of RRD-PVR eyes have been found to contain α-SMA markers for fibroblasts and myofibroblasts, CD45 for hyalocytes, glial fibrillary acidic protein (GFAP) for glial cells, cellular retinaldehyde binding protein (CRALPB) for Müller cells, vimentin for glia and fibroblasts, and Ionized calcium-binding adaptor molecule 1 (IBA1)/CD34/CD68 for microglia [39,73,89,96,102].

Epiretinal membranes from grade C PVR eyes were pigmented and expressed α-SMA, GFAP, Bestrophin-1, and EGF-like module-containing mucin-like hormone receptor-like 1 (F4/80) [13,93]. PVR membranes also contain epithelial membrane protein-2 (EMP2), an integrin regulator [110]. Notch signaling regulates PVR formation and its inhibition reduces α-SMA levels and M2 macrophage infiltration [58]. Macrophages are CD68-positive [39]. PDGF receptors have also been shown to be active in epiretinal membranes [111]. Aquaporin-1 (AQP1) is expressed in the epiretinal membrane in PVR, and it co-localizes with α-SMA and GFAP, suggesting a myofibroblast or glial origin [112]. AQP1 is a protein that promotes cell proliferation and migration [112]. Epidermal growth factor receptor (EGFR) is expressed in early epiretinal membranes [113]. EGFR increases epithelial migration into the wound during re-epithelialization, alters mast cell and neutrophil infiltration, and increases epithelial cell proliferation in early lesions [114]. Epiretinal membranes also contain endothelin receptors A (ETA) and B (ETB ET-1), ET-1 receptors that are upregulated in the vitreous of patients with PVR and RD [50]. ET-1 promotes ocular blood flow, glial proliferation, and collagen contraction [50]. Epiretinal membranes also contain IL-2 receptors, a target of IL-2, which mediates wound healing, e.g., by affecting T lymphocyte development, fibroblasts, and the strength of closed wounds [95,115]. Positive NF-κB labeling has been detected to localize with glial cell markers and IL-8 [116]. Fibrin formation is a common complication of PVR after vitrectomy [117]. Fibrin formation is important for homeostasis, wound healing, and the prevention of bleeding but detrimental in inappropriate scar formation [118,119,120]. Thrombin converts fibrinogen to fibrin monomers, which polymerize of activation by coagulation factor XIII [120]. Plasmin and elastase degrade fibrin and fibrinogen [120]. Plasminogen, an inactive form of plasmin, is increased in the vitreous of PVR eyes, and the ECM acts as a fibrinolytic compound [117]. Plasmin is also known to cause neuroinflammation [121].

In epiretinal membranes, 3194 genes (e.g., alpha-1 type I Collagen [COL1A1], alpha-2 type I Collagen [COL1A2], alpha-1 type III Collagen [COL3A1], fibronectin 1 [FN1], osteonectin/secreted protein acidic and rich in cysteine [SPARC], metallopeptidase inhibitor 1 and 3 [TIMP1/3]) were upregulated in comparison to ILM samples [96]. Circular RNAs (circRNA) have been detected to be dysregulated (55 upregulated and 36 downregulated) in epiretinal membranes in PVR [122]. For example, the most upregulated circRNA is circ_0043144, which indicates a severe disease stage of the disease but decreases after surgery [122]. PDGFA, PDGFC, and KNG1 gene expression were also increased in PVR-epiretinal membranes at the same time as circ_0043144 [122].

**Table 3:** Example list of detected markers in PVR membranes.

Detected Markers in the PVR Membranes		
actin [108]	fibronectin [96,102,107,108,109]	laminin [102,109]
aquaporin-1 (AQP1) [112]	fibroblast-associated markers (antigens CD34, CD44, CD45, CCR7, CXCR4, CCL21, and CXCL12) [63,96,100,102]	metalloproteinases (e.g., matrix metalloproteinase 3 [MMP-3]) [89]
α-SMA [39,63,73,93,96,100,102]	gene dysregulation (e.g., 3194 upregulated, e.g., COL1A1, COL1A2, COL3A1, FN1, SPARC, TIMP1/3) [96]	NF-κB [47,116]
bestrophin-1 [93]	glial fibrillary acidic protein (GFAP) [47,93,102,108]	platelet-derived growth factor (PDGF) A and PDGFC [122]
Immune cell markers (CD4+, CD8+, CD16, CD45, CD45/HLA-DR, CD68, CD163, IBA1/CD206, and CD206) [47,96,102,108]	growth factors (e.g., scatter factor/hepatocyte growth factor family) [89]	PDGF receptors [111]
cellular retinaldehyde binding protein (CRALPB) [102]	hyalocyte-related CD45 [96]	receptor for advanced glycation end products (RAGE) [100]
circular RNA dysregulated (circRNA; e.g., detected to be 55 upregulated and 36 downregulated) [122]	hydrogen peroxide-inducible clone-5 (Hic-5) [100]	secreted protein acidic and rich in cysteine (SPARC)/osteonectin [89,94,96]
collagen [96,102,109]	integrins [123]	Snail [100]
cytokeratin 7, cytokeratin-18/RGE53 [39,94,102]	interleukin-2 receptor [98]	SRY (sex determining region Y)-box 2 (Sox2) [47]
EGF-like module-containing mucin-like hormone receptor-like 1 (F4/80) [93]	interleukin-13 receptor α2 (IL-13Rα2) [100]	tenascin [94,109]
epidermal growth factor receptor (EGFR) [113]	interleukin-8 [116]	thrombospondin 1 (TSP1) [89,94]
epithelial membrane protein-2 (EMP2) [110]	intermediate filaments (e.g., neurofilament) [102]	vimentin [96]
endothelin receptors A (ETA) and B (ETB ET-1) [50]	ionized calcium-binding adaptor molecule 1(IBA1), IBA1/CD206 [96]	
fibroblast activation protein (FAP) [100]	kininogen 1 (KNG1) [122]	

### 2.3. Blood–Retinal Barrier Changes in RRD

The BRB maintains retinal homeostasis by regulating the movement of fluids and molecules, such as oxygen and nutrients, which maintain an optimal retinal microenvironment [124,125,126,127]. The BRBs located at the posterior part of the eye are formed by tight junctions of retinal capillary endothelial cells (inner blood–retinal barrier) and RPE cells (outer blood–retinal barrier), which are in close contact with the Bruch’s membrane and choriocapillaris [124,125,126,127]. In addition to the tight junctions of endothelial cells, pericytes, glia cells, Müller cells, and astrocytes also regulate the function of the inner BRB [126,127]. The outer BRB provides care for the outer layers of the retina, including the RPE cells, photoreceptor outer segments (POSs), external limiting membrane, and outer nuclear layer (ONL) [127]. The inner BRB provides care for the rest of the retina from the ONL to the ILM and all layers in between, including the outer plexiform (PL), inner nuclear layer (INL), inner plexiform (IPL), ganglion layer (GCL), and nerve fiber layer (NFL) [127]. The ILM physically separates the vitreous from the retinal layer and is composed of collagen, laminin, hyaluronic acid, fibronectin, and nidogen [127,128].

Smaller molecules of 20–30 kDa can pass through the BRB, and some small molecules are transported by diffusion [124]. Oxygen and nutrients delivered to the retina are dependent on retinal and choroid blood flow [125]. RRD is associated with vascular leakage, which improves after reattachment [129]. Impaired retinal perfusion may also be associated with RPE atrophy in chronic RRD [130]. In pathological situations, the formation of a subretinal space disrupts the BRB, and RPE cells must remove subretinal fluid into the choroid [2,6,8,30,35]. Microglia are important players in the initiation of the retinal inflammatory response and infiltration of immune cells through the disrupted BRB into the retina from the bloodstream [131]. Both BRBs protect the retina from exogenous antigens, but the breakdown of the BRB during injury (such as RD) causes the infiltration of growth factors and cytokines from retinal blood vessels into the vitreous and subretinal space [80,132].

### 2.4. The Role of Inflammation in the Development of PVR

The immune privilege protects certain vital and non- or weakly regenerative tissues from damage caused by inflammation and foreign antigens [133,134,135,136,137,138]. The BRB protects the retina, but it also has a local immunosuppressive microenvironment maintained both passively and actively [132,133,139]. Typical for the immune privilege is the absence or low levels of major histocompatibility complexes (MHCs), especially MHC I, low antigen presentation capacity, peripheral tolerance to tissue-derived antigens, and the expression of immune suppressants (e.g., TGF-β1) [136,140]. Nevertheless, Zhang et al. showed that retinal microglia express antigen-presenting MHC II [141]. When the BRB is disrupted, antigens enter the retina and activate lymphocyte-mediated immune suppression mechanisms [132]. The antigen enters the systemic circulation and is presented to immune cells that enter the eye and actively suppress inflammation [133,142]. An active component of the immune privilege is vitreous cavity-associated immune deviation (VCAID) [132,137]. VCAID suppresses inflammation in the vitreous cavity, where hyalocytes be antigen-presenting cells [137]. Hyalocytes have been suggested to be phagocytic macrophage-like cells in the vitreous cavity [102,137]. The retina is thought to lack lymphatic vessels, but they are present, for example, in the choroid [139,140]. It is now suggested that the posterior segment of the eye also has a lymphatic system [143,144]. For example, samples of the pre-macular bursa collected from macular hole patients expressed lymphatic vessel endothelial hyaluronan receptor 1 (LYVE-1) [145]. Lymphatic endothelial vessels have also been observed in the rat retina in the outer and inner plexiform layers, which are connected to radial blood vessels [144]. The concept of immune privilege retina has therefore been questioned when highly controlled immunity would be more appropriate [146].

Peripheral monocytes and neutrophils are the most common leukocytes that infiltrate the retina and subretinal space during retinal detachment, contributing, e.g., to photoreceptor degeneration [147]. Complement activation, microglia, Müller cells, astrocytes, and endothelial cells are all involved in the retinal immune responses in RD [132]. Of the proteins measured, 60% have been suggested to be contained in exosomes, implying secretion from nearby cells into the vitreous [26,65]. Exosomes are a major component of the vitreous, promote cell–cell and vitreoretinal communication, and contain proteins that influence immunity, immune tolerance, and antigen-presentation [65,148]. The size and depth of the retinal detachment contribute to the increase in IL-8, TGF-β3, TIMP-1 and 2, myeloperoxidase (MPO), inducible protein 10 (IP-10), and vascular cell adhesion molecule 1 (VCAM-1) and decrease in neural cell adhesion molecule (NCAM) levels in the vitreous of RRD patients compared to controls in idiopathic epiretinal membrane cases [149].

Josifovska et al. showed that subretinal fluid samples of RD patients have elevated levels of at least 38 cytokines (e.g., angiogenin, RBP-4, cystatin C, apolipoprotein A 1, C-reactive protein, vitamin D BP, TIM-3, IL-18 Bpa, chitinase 3-like 1, SHBG, VCAM-1, IGFBP-2, BAFF, emmprin, osteopontin, adiponectin, CD14, complement component C5/C5a, DPPIV, CD31, HGF, lipocalin-2, serpin E1, IL-8, MCP-1, GDF-15, endoglin, complement factor D, ICAM-1, uPAR, Dkk-1, BDNF, FGF-19, Flt-3 ligand, macrophage migration inhibitory factor (MIF), SDF-1α, vascular endothelial growth factor [VEGF], and IGFBP-3) [47]. Growth factors and cytokines (e.g., TGF-β, PDGF, TNF-α, IL-6, and IL-8) have been suggested to be critical factors in the development of PVR [150]. Cytokine levels (CCL27, CXCL6, IL4, IL16, CXCL10, CCL8, CCL22, MIG/CXCL9, CCL15, CCL19, CCL 23, and CXCL12) are elevated in the vitreous humor of PVR patients, depending on the stage of the disease [46]. CCL19 is suggested to be an early biomarker [45]. Eastlake et al. showed that PVR retinas have increased levels of growth factors and cytokines (e.g., TFF3, I-TAC, IL-16, IL 18Bpa, DPPIV, uPAR, IL-1a, PF4, macrophage inflammatory protein [MIP-3b], TfR, VEGF, IL-23, M-CSF, adiponectin, IL-2, ENA-78, PDGF-AB/BB, SDF-1a, pentraxin-3, kallikrein, resistin, aggrecan, angiogenin, C-reactive protein, MMP-9, MPO) compared to normal retinas [82]. The inflammatory cytokines GM-CSF, G-CSF, RANTES, MCP-1, IL-9, IL-17A, IL-12, IL-RA, IL-10, MIP-1B, IL-8, IL-6, IL-13, IL-2, IL-4, MIP-1α, and IL-1β were also found to be significantly increased in the gliotic retinal samples of PVR patients who underwent retinectomy compared to cadaveric retina [82].

The levels of IL-6 and interferon (IFN)-ɣ were twice as high in RRD eyes, and IFN-ɣ was six times as high in the subretinal fluid of PVR patients compared to cadaveric controls [151]. For example, TGF-β2 and bFGF are higher in the vitreous of pre and post-operative PVR groups compared to controls [152]. Pre-PVR is associated with higher IL-1β and post-PVR with higher IL-6 levels than controls [152]. High vitreous protein and IL-6 levels were interpreted as significant risk factors for post-operative PVR formation [152]. Limb et al. showed that IL-1 and IL-6 concentrations were high in the vitreous of RD and PVR eyes compared to cadaveric controls [153]. They suggested that IFN-ɣ was more prevalent in the vitreous of PVR patients than in patients with retinal detachment without PVR or the vitreous of control cadavers, while, unexpectedly, TGF-β did not differ significantly between groups [153]. Balogh et al. showed that, in the vitreous fluid, the levels of IL-6, IL-8, IL-16, MCP 1, eotaxin, IFN-ɣ, MIF, and MIP-1 beta were increased in RRD eyes with or without PVR [154]. Wang et al. reported that, after retinal detachment, MCP-1 secretion is increased when monocytes/macrophages infiltrate the vitreoretinal space, and the infiltrated monocytes secrete IL-6 instead of retinal cells [52]. Fibroblast proliferation activity (FPA) was higher in the vitreous control cadavers than in patients with retinal detachment or PVR [153]. Many of the activated pathways by vitreous proteins are linked to the Wnt and MAPK signaling pathways [26].

## 3. Wound Healing Process

### 3.1. General Aspect to Acute Wound Healing Phases

**Figure 2:**
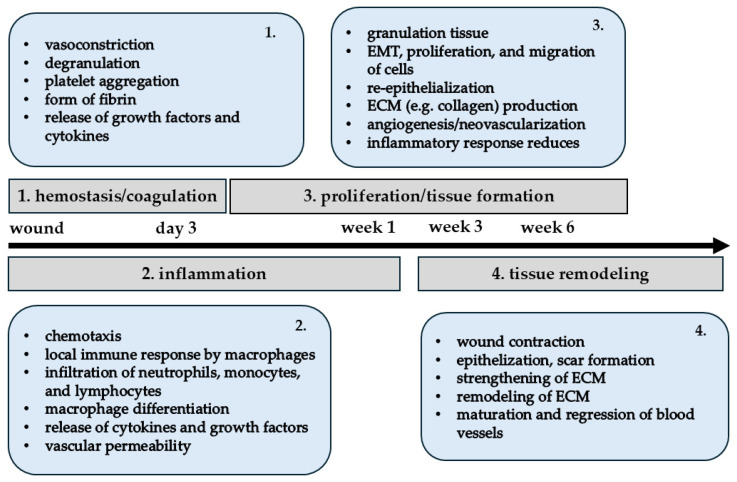
A general description of the wound healing timeline.

Normally, wounds heal (e.g., skin) within 4 to 6 weeks (Figure 2) and include hemostasis, inflammation, proliferation, and remodeling phases, all of which are well controlled, continuous, and sequential but also overlapping [155,156,157]. Hemostasis involves vasoconstriction, degranulation, platelet aggregation, and the formation of a fibrin clot that seals the wound [155,158]. The coagulation process refers to platelets surrounded by cross-linked fibrin, which is formed by the cleavage of fibrinogen by thrombin [158]. One of the earliest cellular signals following injury is calcium, which triggers NADPH oxidase to produce H_2_O_2_, which recruits leukocytes [159,160]. In addition, cytokines such as IL-1α and IL-33 are stored intracellularly and released as danger signals during wounding [158]. In the first phase, platelets, wounds, and clots release, for example, growth factors (platelet factor 4, TGF-β, PDGF, FGF, epidermal growth factor [EGF], and VEGF-A), pro-inflammatory cytokines/chemokines (e.g., IL-8 and MIP2-alpha/CXCL2), and danger-associated molecular patterns (DAMPs; e.g., ATP and high-mobility group Box 1 protein [HMGB-1]) [155,156,158,161]. Platelets release serotonin and histamine, which promote cell permeability [157].

When blood flow is regulated in the first phase, the inflammation phase begins, and a rapid macrophage response and the chemotaxis-related infiltration of neutrophils, monocytes, and T lymphocytes as well as the differentiation of macrophages are triggered [155,158,161]. Resident and infiltrated macrophages respond to factors released from damaged tissue and begin to regulate damage and inflammation [162]. Macrophages release factors (e.g., TNF-α, TGF-β, interleukins, Wnt, FGF, VEGF, PDGF, IGF) that regulate the activation, differentiation, and proliferation of endothelial and epithelial cells, fibroblasts, and progenitor and stem cells to support the inflammatory response and the healing process [162]. Once healing is complete, macrophages switch to a differentiated phenotype, reducing inflammation and repairing structure [162]. If the macrophage response is misdirected, inflammation persists, increasing susceptibility to fibrosis [162]. Macrophage activity can be pro- or antifibrotic [163]. In the early stages of wound healing, macrophages express IL-1, IL-6, TNF-α, ROS, and MMPs, and, during prolonged healing, also PDGF, IGF-1, VEGF-A, TGF-β, TIMP1, and anti-inflammatory IL-10 [161]. IL-4 levels also increase during the inflammatory phase [156]. Macrophages activate and recruit fibroblasts and inflammatory cells (e.g., by releasing TGF-β and PDGF), regulate MMPs, TIMPs, and fibrogenesis by releasing cytokines and are important regulators of fibrosis [163]. Neutrophils proliferate in the wound and participate in healing, e.g., by clearing debris and microbes, performing phagocytosis, producing ROS, releasing antibacterial granules and cytokines (IL-1, IL-6, TNF-α), and forming neutrophil extracellular traps (NETs) [155,161].

During the proliferation phase, granulation tissue and new epithelium are formed (re-epithelialization), the ECM is produced (e.g., collagen), angiogenesis is induced, inflammation is reduced, and mesenchymal cell differentiation, proliferation, and migration are increased [155,158,161]. Granulation tissue fills the wound, is contractile, and consists of fibroblasts, myofibroblasts, inflammatory cells, endothelial cells, ECM components, and new capillaries [161,164,165]. During the proliferation phase, fibroblasts proliferate, produce ECM deposits, and secrete, among others, proteinase, VEGF, and FGF [156]. Fibroblasts migrate to the wound in 3–4 days, attracted by TGF-β, PDGF, and FGF and differentiate into myofibroblasts [156]. When the wound is closed, the final remodeling phase begins [164]. It includes wound contraction, epithelization, tissue reinforcement by fibroblast-mediated collagen synthesis, collagen and ECM remodeling, vascular maturation and regression, and scar formation [155,156,161]. Wound healing is characterized by cell proliferation and migration, as well as structural re-organization and ECM production with connections to the surrounding tissue [166]. Inflammation is a response to injury and is required for repair, but wounds that do not heal properly remain in the inflammatory phase [155,167].

The ECM provides the basic structure of tissues but is dynamic, self-renewing, and remodeled constantly in contact with the surrounding cells [164,168]. The ECM can bind to cell receptors (e.g., integrins), providing biochemical rigidity, and influencing cell signaling, including the release and storage of growth factors and signaling molecules (e.g., TGF-β, amphiregulin, Wnt, EGF, and FGF) [168,169,170]. Integrin interactions can cause, e.g., the activation of Smad, Akt, and Erk1/2 signaling [169]. Changes in the amount, composition, and structure of the ECM can promote fibrosis [168]. The concentration gradient of components in the ECM affects cell migration and distribution [164,170,171]. The ECM is elastic and degraded by migrating cells, and it regulates cell differentiation and signaling through receptors [170]. Carbohydrate complexes, immune components (e.g., cytokines, mucins, complement factors, vitronectin), growth factors, extracellular proteases (e.g., MMP), lectins, galectins, and remodeling regulators are present in the ECM [168,172].

Fibroblasts enter the ECM during wound healing, differentiate into TGF-β-induced myofibroblasts, and produce the ECM [164]. TGF-β is an important transcription factor in wound healing, i.e., in inflammation, neovascularization, granulation tissue formation, re-epithelization, fibrosis, and cell proliferation and differentiation [173,174,175]. Normally, TGF-β functions as a cytokine or growth factor to maintain homeostasis [174]. In wound healing, TGF-β induces beneficial ECM-associated collagen production, which is detrimental if continued as fibrosis [68,176]. TGF-β is activated after injury, for example by integrins, leading to the activation of Smad and EMT factors (e.g., fibronectin, α-SMA, and collagen) and the secretion of cytokines and growth factors [68,174,176,177,178,179]. Integrins connect the ECM to the cell cytoskeleton, regulate the connection to blood vessels, and promote cell proliferation, migration, adhesion, and survival [168,170,172,180]. Integrin association with the ECM is dynamic, causing cell movement [170]. Proteases, such as MMP, also facilitate cell migration by degrading the ECM [168,170].

#### Wound Healing and Fibrosis

Adults form scar tissue during wound healing, whereas the fetus has the ability to heal without inflammation and scarring [164]. Many local and systemic factors influence wound healing including hypoxia, ischemia, bacterial infection, age, gender, hormones, stress, diseases, obesity, nutrition, medications, and lifestyle [155,157]. Optimal oxygen levels are important for proper wound healing, as hypoxia induces the secretion of growth factors, and local ischemia is typical for surgical wounds [155,157,161]. Fibrinogenesis is a normal response after injury, but, in combination with inflammation, it leads to fibrosis [181]. Inflammation is important for early wound healing, but, when prolonged, it can be a cause of fibrosis [164].

Fibrosis is the overgrowth and hardening of scar tissue [74,92]. Fibrosis can be divided into well-known stages, including the occurrence of injury, the inflammatory response, and the activation and differentiation of α-SMA-positive myofibroblasts from fibroblasts, pericytes, and other local cells to produce the ECM [74]. Fibrosis is a defective wound healing process [74,168]. One critical factor in scar formation is the conversion of collagen 3 to collagen 1 [156]. In controlled repair, the ECM and tissue are repaired to baseline level [74]. In fibrosis, the ECM is overproduced and organized in an uncontrolled manner, resulting in a disorganized and rigid structure that causes the abnormal function of the diseased tissue [74,181,182]. The ECM contains a reservoir for growth factors that bind to heparin and heparan sulfate, from which growth factors can be activated, distributed, and presented to cells as needed, e.g., in morphological cell changes and proliferation [168,171].

Myofibroblasts are key influencers in fibrosis because they overproduce ECMs, particularly collagen, elastin, and proteoglycans [92,167,175]. Myofibroblasts are transformed through EMT from epithelial cells, endothelial cells, and local mesenchymal cells [92]. TGF-β induces fibroblasts to become active myofibroblasts, characterized by the increased expression of α-SMA, collagen, and actin-related proteins [175]. Autocrine signals, macrophages, lymphocytes, and fibroblasts activate myofibroblasts [92]. Molecules that contribute to fibrosis include IL-13, IL-21, IL-33, TGF-β1, MCP-1, caspases, MIP-1β, VEGF, PDGF, peroxisome proliferator-activated receptors (PPARs), and acute phase proteins (SAP) [92,168]. There are three types of fibrogenic cells in damaged tissue, including fibroblast-like cells/endogenous fibroblasts, EMT cells, and, finally, recruited fibroblasts [183].

In fibrosis, TGF-β1 and TGF-β2 levels are increased, but TGF-β3 is decreased, fibroblasts, myofibroblasts, and TIMPs are increased, while MMPs are reduced [164]. MMPs are major enzymes of the ECM, which are increasingly activated during repair, but also during the disease process as inflammation, while TIMPs can deactivate MMPs [74,168]. MMPs are mostly extracellular enzymes that degrade the ECM, preventing fibrosis, but they have also been reported to promote fibrosis, among others [74]. MMP-1 and 2 have anti-fibrotic features, and MMP-12 and 13 have pro-fibrotic properties [74].

### 3.2. Wound Healing and Fibrosis in the Retina

**Figure 3:**
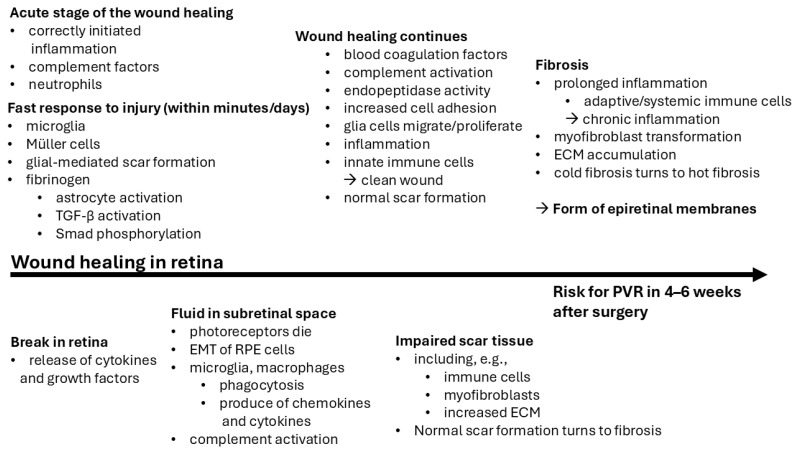
Wound healing in the retina.

After RRD surgery, the risk for developing PVR most commonly occurs within 4–6 weeks (Figure 3), but, overall, the first three months are the risk period [14,184]. The retinal wound healing process is very similar to that in the central nervous system (CNS) [185]. Glial cells play an important role in CNS-related wound healing and are comparable to fibroblasts in normal non-CNS-related wound healing [185]. Wound healing in RRD has been demonstrated by increased blood coagulation factors (coagulation factor V and von Willebrand factor), complement activation, endopeptidase activity (e.g., TIMP1), as well as increased cell adhesion and scar formation [26]. TGF-β protects retinal neurons during injury, for example, by protecting retinal ganglion cells (RGCs) by reducing ROS and increasing antioxidant signaling (e.g., HO-1 and Nrf-2) [186]. Retinal break causes RPE cell contact with cytokines and growth factors released from the ECM or the vitreous, leading to RPE cell proliferation and migration and, ultimately, the formation of contractile epiretinal membranes [150]. The retinal ECM is divided into two components: the interphotoreceptor matrix (IPM) and the rest of the retinal ECM [172].

Properly initiated inflammation, complement factors, neutrophils, and glial cells are responsible for the initial phase of wound healing [187]. For example, glial cells migrate to the vicinity of the wound [187,188]. However, if healing is complicated, the induced inflammation leads to pathological fibrosis [187]. In the damaged retina, glial cells proliferate in the retinal wound area, and the accumulation of monocytes triggers the induced inflammation [189]. When the induced inflammation is successfully regulated and suppressed in a timely manner, the formation of epiretinal membranes is prevented [189]. When the initial inflammation subsides, the retinal wound is closed, and leukocyte infiltration is prevented, the healing process ends in the gliosis (“cold fibrosis”) [187]. In the acute phase, innate immune cells clean the wound, but, if prolonged, adaptive immune cells (T and B lymphocytes) arrive, and the healing process changes to “hot fibrosis”, including myofibroblast transformation and ECM accumulation [187]. Miller et al. proposed that acute inflammation and the macrophage response are key factors in retinal wound healing, scar formation, and epiretinal membrane formation [189]. After vitrectomy, retinal scars are irregular and hypertrophic compared to non-vitrectomized eyes, in which retinal wounds heal better, forming regular and smooth scars, because the vitreous plays a role in retinal wound healing [190]. During wound healing in the subretinal space between photoreceptors and RPE cells, retinal microglia and complement activation are the first defense mechanisms to be activated [187]. If healing is prolonged, systemic immune cells are involved in the healing process, which is an undesirable response and can lead to a chronic inflammatory response with retinal and subretinal fibrosis [187]. If the immune system does not support healing, the scar tissue contains immune cells, myofibroblasts, and an excess ECM [92]. If photoreceptors die as a result of injury, it can lead to the loss of immune privilege of the subretinal space and the EMT of RPE cells [92]. Microglia and macrophages enter the damaged subretinal space to phagocytosis and produce chemokines and cytokines [92]. In addition, phagocytic RPE cells and Müller cells are activated in healing [92]. After clearance, innate immunity heals the remainder of the lesion, but, if the lesion is large, ECM and Müller cells fill the space, forming gliosis [92].

Myofibroblast balance is important for both proper wound healing and the prevention of excessive scar formation [191]. Myofibroblasts can be differentiated from macrophages, Müller cells, and RPE cells [92,187]. Neutrophils are the first line of defense in acute injuries, but macrophages also participate in healing by cleaning debris [187]. Macrophages secrete pro-angiogenic and fibrotic components, induce EMT, and recruit fibroblasts, while retinal microglia act as phagocytic cells in the injured neuronal area [187,192].

Epiretinal membrane formation associated with PVR is an abnormal wound healing process in which cytokines and growth factors induce cell proliferation (e.g., RPE cells, astrocytes, Müller cells) [113]. Formed epiretinal membranes include myofibroblast-like cells that express α-SMA [193]. It is noteworthy that RRD with vitreous hemorrhage predicts a higher risk for developing PVR [21,187]. Retinal fibrosis disrupts normal vision and causes the dysfunction of normal retinal tissue [185]. PVR formation represents the final stage of impaired wound healing, i.e., the scarring [15]. Proper TGF-β signaling is important in the eye, and abnormalities in TGF-β signaling are an important part of abnormal angiogenesis, neurogenesis, inflammatory responses, and fibrosis [194]. The TGF-β family consists of three isoforms, TGF-β1, TGF-β2, and TGF-β3, from which TGF-β2 and TGF-β3 are present in RPE cells, TGF-β1 and TGF-β2 in photoreceptor outer segments, and TGF-β3 in photoreceptor inner segments [195,196]. In the eye, TGF-β2 is proposed to be the most prominent isoform [197]. Injury leads to increased levels of TGF-β, IL-13, and the connective tissue growth factor (CTGF), causing the activation of fibroblasts and myofibroblasts that produce more ECMs and lead to fibrosis if not balanced by degradation [168]. TGF-β levels were increased in the vitreous of RRD with PVR compared to retinal detachment without PVR [198]. TGF-β levels increased concomitantly with the progression of PVR [198]. In rabbit eyes, TGF-β with fibronectin increased fibrosis, whereas TGF-β alone did not [198]. TGF-β induced fibroblasts to differentiate into myofibroblasts that express α-SMA with the co-adhesion of integrin and fibronectin affecting contractile properties and cell migration [175,199].

Impaired angiogenesis and hypoxia (e.g., hypoxia-inducible factor-1 alpha [HIF-1α]) are characteristic features of fibrosis [162]. In PVR, the ECM is over-produced and forms fibrosis, while, in geographic atrophy, the injured space is filled with Müller cells and infiltrated immune cells rather than ECM [92]. RPE cells can become either fibroblast-like cells in an ECM-rich environment or macrophage-like cells near the photoreceptor or vitreous [92]. Although macrophages are important at the initiation of wound healing, they can cause inflammation-related fibrosis at a later stage of healing [187]. Macrophages are pro-fibrotic, induce angiogenesis, undergo macrophage-myofibroblast transition, induce RPE and endothelial cell EMT, recruit fibroblasts, and promote inflammation [187]. Mast cells contribute to epiretinal membrane formation and angiogenesis [187]. In fibrovascular membranes, the level of IL-17 produced by T lymphocytes is increased [187]. Müller cells are more resistant to damage than neurons due to their specialized energy metabolism, antioxidant capacity, and regenerative and proliferative capacity [200]. Müller cells migrate to the outer retina during retinal detachment [200].

Fibrosis causes pathobiological changes associated with injury in the retina [185]. Local responses during injury include permeability changes in blood vessels, inflammation, immune cell infiltration, neovascularization, fibroblast infiltration and proliferation, and ECM changes [185]. Vitreous hemorrhage can trigger the proliferation of hyalocytes and monocytes that can migrate to the retina [201]. Microglia can migrate from the retina to the vitreous due to hemorrhage [201]. The proliferative cells in the damaged retina have migratory properties from the outer retina to the vitreous direction and the properties to be phagocytic [201]. Müller cells, astrocytes, RPE cells, and glial cells form the glial scar, also known as sub- and epiretinal membranes, which have been observed in the vitreous, adjacent to the ILM, and in the subretinal space [200,201].

#### Glial Scar Formation

Damage to the CNS induces gliosis and can lead to the formation of glial scars, which includes the accumulation of cells, e.g., microglia, astrocytes, oligodendrocytes, meningeal cells, and stem cells [202,203]. Gliosis attempts to protect neurons and prevent damage but becomes detrimental when it leads to scarring [204]. Glial scars fill the damaged area of neurons, photoreceptors, RPE cells, and blood vessels forming new tissue on both sides of the retina [204]. Gliosis is a similar kind of process in the CNS and neuroretina except for retinal Müller cells, which are the principal glial cells only in the retina [204,205]. Epiretinal membranes are a type of formed glial scar between the vitreous and the ILM [200]. Glial cells support cells for neuronal tissue and do not participate in neuronal transport or synaptic activity [192]. The retina has three types of glial cells: astrocytes, Müller glia, and microglia [206]. Macroglia cells (Müller cells and astrocytes) support retinal structure and homeostasis [200,206]. For example, macroglia secrete growth factors and regulate glucose, neurotransmitter, and ion homeostasis, support blood circulation, participate in waste removal, support the BRB, protect against ROS, and mediate local immune responses [200]. Glial cells respond to retinal homeostatic changes through gliosis, which is a neuroprotective response but also a chronically harmful one that reduces regeneration [200,203]. Müller cells are usually the first glial cells to respond to damage due to distribution across neuroretina [200,204]. Macroglia migrate to and cover the wound, eliminate pathogens, support neuronal survival, secrete cytokines and chemokines, and activate antioxidant defense mechanisms and phagocytosis-related enzymes [200]. Astrocytes are located especially near ganglion cells and respond to injury by expressing GFAP and hypertrophy and by acquiring stem cell properties [200,207]. Microglia are resident immune cells that secrete cytokines, chemokines, and growth factors and perform the phagocytosis of pathogens, dead cells, and protein aggregates being activated within minutes after injury [208,209]. Müller cells migrate to the damaged area and fill the gap caused by the injury by replacing the damaged photoreceptors and neurons [200]. Elongated Müller cells extend from the ILM to the outer limiting membrane, thus being the link between neurons, blood vessels, the vitreous, and the subretinal space [200]. At the beginning of wound healing, Müller cells secrete neuroprotective compounds (e.g., VEGF), thus increasing survival [200].

In active gliosis, glial proliferation and migration, cellular hypertrophy, and increased levels of GFAP, vimentin, and nestin occur [200]. Proliferating Müller cells do not express GFAP in the damaged retina, and cells expressing GFAP do not proliferate [204,210]. Müller cells express intermediate filament nestin during gliosis-associated scar formation [211]. Synemin-labeled and activated astrocytes are also present in the damaged retina [211,212]. The nestin of Müller cells and synemin of astrocytes network with GFAP and vimentin [200,212]. Retinal Müller cells ensheathe retinal neurons and support homeostasis [213]. Müller cells secrete neurotrophic factors, erythropoietin, cytokines, and growth factors, but they also assist in neovascularization, have antioxidant properties (e.g., release glutathione), and uptake glutamate, which is neurotoxic to the inner retina [204,213]. Gliotic Müller cells have lost their phenotype to protect neurons, e.g., due to impaired ion balance leading to swelling and neuronal death [213].

TGF-β and Smad-related CNS-type glial scar formation begins in a few hours after injury, and an early marker is leaking fibrinogen from the broken BRB [214]. Fibrinogen is an astrocyte activator that triggers the formation of glial scars and whose inhibition prevents glial activation, due to decreased TGF-β activity and Smad phosphorylation [214]. TGF-β2 is increased in the gliotic retina, whereas TGF-β1 and TGF-β3 levels were not different compared to the control [82]. After a few days of injury, microglia cells appear in the damaged area for phagocytosis [210]. At the same time, mitotically active Müller cells become progenitor cell phenotypes and migrate to the inner and outer nuclear layers [204,210]. Müller cells have been suggested to have progenitor cell properties through dedifferentiation and potential to regenerate damaged neuronal tissue [200,204,215]. For example, glutamate signaling and α-aminoadipate have been proposed to stimulate the migration of Müller cells into the outer nuclear layer and transform to neurons and photoreceptors [204,215]. Müller cells migrate via the intermediate filament to the outer retina during damage, proliferate, participate in gliosis, and are present in the subretinal scar [216].

In hypertrophic gliosis, the BRB breaks, and cytokines, growth factors, and infiltrated immune cells increase [204]. Gliosis that secretes neurotrophic factors and increases glutamate transporter proteins and intraretinal glutamate levels could be protective [204]. Müller cell proliferation is characteristic in PVR-related gliosis and is suggested to be a critical inflammatory mediator in the process, for example, by increasing IL-2, IL-16, IL-1a, VEGF, uPAR, adiponectin, TFF3, DPPIV, PF4, TfR, M-CSF, PDGF-AB/BB, and SDF-1a [81]. Müller cells secrete in vitro, for example, TGF-β2, TNF, VEGF, MCP-1, G-CSF, PDGF-bb, NO, and RANTES [82,204]. In the gliotic retina, MMP-9, MPO, aggrecan, angiogenin, and c-reactive protein were reduced when IL-1α, IL-2, IL16, VEGF, adiponectin, TFF3, DPPIV, uPAR, PF-4, TfR, M-CSF, SDF-1α, I-TAC, IL-18Bpa, MIP-3b, IL-23, and ENA-78 were increased in comparison to the normal retina [82].

## 4. Conclusions

Scar formation associated with retinal wound healing is undesirable after RRD surgery. Inflammatory responses and EMT, proliferation, and migration of the retinal cells are important targets to prevent fibrosis associated with PVR formation. It is important to develop new methods to prevent the formation of fibrotic membranes, thus supporting recovery from the RRD surgery. To achieve this, it is necessary to study the role of different cells and molecules in the formation of epiretinal membranes using isolated membranes from the eyes of PVR patients. Also, preventive treatment compounds (i.e., statin implants) and potential combinatory treatments against scar membrane formation need to be studied as well as the importance of EMT and its regulation in the retinal wound healing process.

## Figures and Tables

**Table 1:** Example of increased proteins in the vitreous of PVR eyes compared to acute RRD [26].

Group of Protein	Proteins
cell adhesion molecules	plakophilin-1 opioid-binding protein annexin A2 alpha-actinin-1
apoptotic-related proteins	protein S100-A14 fructose-2,6-bisphosphatase TIGAR
signaling molecules	proteasome subunit beta type-1 growth factor receptor-bound protein 2 pseudokinase FAM20A protein S100-A11 copine-1
retinol transporter	transthyretin (prealbumin)
other	SUMO-activating enzyme subunit 1 arginine-tRNA ligase rod cGMP-specific 3′,5′-cyclic phosphodiesterase subunit alpha

**Table 2:** Cells in PVR membranes.

Detected Cell Types in the PVR Membranes After RRD	
astrocytes [39,93,96]	Müller cells [39]
fibroblasts/myofibroblasts [39,64,93,96,97]	myeloid cells [96]
hyalocytes [96]	Myo/Nog cells [97]
macrophages [39,47,93,95,96]	preadipocytes [96]
melanocytic cells [96]	progenitor and stem cells [47]
microglia [39,47,93,96]	RPE cells [39,93,94,96]
monocytes [95]	T lymphocytes [95]

## References

[1] Moysidis S.N., Thanos A., Vavvas D.G. (2012). Mechanisms of Inflammation in Proliferative Vitreoretinopathy: From Bench to Bedside. Mediat. Inflamm..

[2] Lalezary M. (2015). Vanishing Retinal Detachment. Retin. Cases Brief Rep..

[3] Zhang L., Chen B., He W. (2023). Occult Intraocular Aluminium Foreign Body Causing Rhegmatogenous Retinal Detachment: A Case Report. BMC Ophthalmol..

[4] Polkinghorne P.J., Craig J.P. (2004). Analysis of Symptoms Associated with Rhegmatogenous Retinal Detachments. Clin. Exp. Ophthalmol..

[5] Tsui J.C., Brucker A.J., Kolomeyer A.M. (2024). Rhegmatogenous Retinal Detachment with Concurrent Choroidal Detachment and Macular Hole Formation after Uncomplicated Cataract Extraction and Intraocular Lens Implantation: A Case Report and Review of Literature. Retin. Cases Brief Rep..

[6] Abrishami M., Smith S.M., Slomovic A.R., Altomare F., Krema H. (2024). Rhegmatogenous Retinal Detachment Associated with an Epibulbar Tumour. Can. J. Ophthalmol..

[7] Boral S., Agarwal D., Mohanta A. (2022). Rhegmatogenous Retinal Detachment Following Femtosecond Laser-Assisted Cataract Surgery. Oman J. Ophthalmol..

[8] Wang J.C., Qian C.X., Comander J.I. (2022). Repair of Rhegmatogenous Retinal Detachment in Choroideremia Secondary to Posterior Extramacular Retinal Hole. Ophthalmic Surg. Lasers Imaging Retin..

[9] Samanta R., Sood G., Waghamare S.R., Mareguddi R.R., Mittal S.K., Agrawal A. (2020). Management of a Unique Case of Post-Traumatic Posterior Giant Retinal Tear and Macular Hole-Associated Rhegmatogenous Retinal Detachment. Indian J. Ophthalmol..

[10] Wendt S.P., Barrett D.A., Chang E.Y., Schefler A.C. (2021). Novel Insights from Clinical Practice Segmental Scleral Buckle Surgical Technique for Repair of a Rhegmatogenous Retinal Detachment in Retinoblastoma: A Case and Review of the Literature Established Facts. Ocul. Oncol. Pathol..

[11] Benmerzouga Mahfoudi N., Chaker Harbi M., Boulaneb Beddiar F., Chachoua L. (2015). Bilateral Retinal Detachment and High Myopia: Report of Nine Cases. J. Fr. Ophtalmol..

[12] Sotelo-Monge K.G., Muñoz Escudero M., Ayala Fuentes M.E., Puntí Badosa A., Anton A. (2023). Increased Intraocular Pressure Secondary to Retinal Detachment. Arch. Soc. Esp. Oftalmol. (Engl. Ed.).

[13] Hilton G., Machemer R., Michels R., Okun E., Schepens C., Schwartz A. (1983). The Classification of Retinal Detachment with Proliferative Vitreoretinopathy. Ophthalmology.

[14] Idrees S., Sridhar J., Kuriyan A.E. (2019). Proliferative Vitreoretinopathy: A Review. Int. Ophthalmol. Clin..

[15] Pastor J.C. (1998). Proliferative Vitreoretinopathy. Surv. Ophthalmol..

[16] Pastor J.C., de la Rúa E.R., Martín F. (2002). Proliferative Vitreoretinopathy: Risk Factors and Pathobiology. Prog. Retin. Eye Res..

[17] Tseng W., Cortez R.T., Ramirez G., Stinnett S., Jaffe G.J. (2004). Prevalence and Risk Factors for Proliferative Vitreoretinopathy in Eyes with Rhegmatogenous Retinal Detachment but No Previous Vitreoretinal Surgery. Am. J. Ophthalmol..

[18] Girard P., Mimoun G., Karpouzas I., Montefiore G. (1994). Clinical Risk Factors for Proliferative Vitreoretinopathy after Retinal Detachment Surgery. Retina.

[19] Lleó Pérez A., Campos Fernández R., López Santoveña F., Sánchez Lorente G., Hernández Martínez F.J., Navarro Palop C. (2000). Clinical Risk Factors for Proliferative Vitreoretinopathy after Retinal Detachment Surgery. Arch. Soc. Esp. Oftalmol..

[20] Xiang J., Fan J., Wang J. (2023). Risk Factors for Proliferative Vitreoretinopathy after Retinal Detachment Surgery: A Systematic Review and Meta-Analysis. PLoS ONE.

[21] Yeung L., Yang K.-J., Chen T.-L., Wang N.-K., Chen Y.-P., Ku W.-C., Lai C.-C. (2008). Association between Severity Ofvitreous Haemorrhage and Visualoutcome in Primaryrhegmatogenous Retinaldetachment. Acta Ophthalmol..

[22] Lewis G.P., Charteris D.G., Sethi C.S., Leitner W.P., Linberg K.A., Fisher S.K. (2002). The Ability of Rapid Retinal Reattachment to Stop or Reverse the Cellular and Molecular Events Initiated by Detachment. Investig. Ophthalmol. Vis. Sci..

[23] S F Geller G.P.L.D.H.A.S.K.F. (1995). Use of the MIB-1 Antibody for Detecting Proliferating Cells in the Retina. Investig. Ophthalmol. Vis. Sci..

[24] Asaria R., Gregor Z. (2002). Simple Retinal Detachments: Identifying the at-Risk Case. Eye.

[25] Yang S., Li H., Li M., Wang F. (2015). Mechanisms of Epithelial-Mesenchymal Transition in Proliferative Vitreoretinopathy. Discov. Med..

[26] Öhman T., Gawriyski L., Miettinen S., Varjosalo M., Loukovaara S. (2021). Molecular Pathogenesis of Rhegmatogenous Retinal Detachment. Sci. Rep..

[27] Blair K.C.C. (2024). Retinal Detachment. StatPearls [Internet].

[28] Zhao X., Huang L.I., Lyu C., Liu B., Ma W., Deng X., Jiang H., Wang Y., Yu X., Ding X. (2020). Comparison between Releasable Scleral Buckling and Vitrectomy in Patients with Phakic Primary Rhegmatogenous Retinal Detachment. Retina.

[29] Fisher S.K., Lewis G.P., Linberg K.A., Verardo M.R. (2005). Cellular Remodeling in Mammalian Retina: Results from Studies of Experimental Retinal Detachment. Prog. Retin. Eye Res..

[30] Kohmoto R., Fukumoto M., Sato T., Oosuka S., Kobayashi T., Kida T., Suzuki H., Ikeda T. (2019). Rhegmatogenous Retinal Detachment with a Giant Tear Located in the Intermediate Periphery Two Case Reports. Medicine.

[31] Mishra C., Tripathy K. (2023). Retinal Traction Detachment. StatPearls [Internet].

[32] Tsiogka A., Karamaounas A., Papakonstantinou E., Petrou Sr P. (2021). Tractional Retinal Detachment in a Patient With Waldenström’s Macroglobulinemia. Cureus.

[33] Bilgic A., Sudhalkar A. (2022). Minimal Surgery for Tractional Retinal Detachment Secondary to Branch Retinal Vein Occlusion: A Case Report. J. Med. Case Rep..

[34] Ratanasukon M., Wongchaikunakorn N. (2005). Exudative Retinal Detachment after Photodynamic Therapy: A Case Report in an Asian Patient. Eye.

[35] Amer R., Nalcı H., Yalçındağ N. (2017). Exudative Retinal Detachment. Surv. Ophthalmol..

[36] Simunovic M.P., Shao E.H., Osaadon P. (2020). Ab-Externo Drainage with Continuous Anterior Chamber Infusion for Non-Resolving Exudative Retinal Detachment: A Case Report. BMC Ophthalmol..

[37] Ge J.Y., Teo Z.L., Chee M.L., Tham Y.-C., Rim T.H., Cheng C.-Y., Wong T.Y., Wong E.Y.M., Lee S.Y., Cheung N. (2024). International Incidence and Temporal Trends for Rhegmatogenous Retinal Detachment: A Systematic Review and Meta-Analysis. Surv. Ophthalmol..

[38] Feltgen N., Walter P. (2014). Rhegmatogenous Retinal Detachment-an Ophthalmologic Emergency. Dtsch. Arztebl. Int..

[39] Mudhar H.S. (2020). A Brief Review of the Histopathology of Proliferative Vitreoretinopathy (PVR). Eye.

[40] Chaudhary R., Dretzke J., Scott R., Logan A., Blanch R. (2016). Clinical and Surgical Risk Factors in the Development of Proliferative Vitreoretinopathy Following Retinal Detachment Surgery: A Systematic Review Protocol. Syst. Rev..

[41] Kon C.H. (2000). Risk Factors for Proliferative Vitreoretinopathy after Primary Vitrectomy: A Prospective Study. Br. J. Ophthalmol..

[42] Bonnet M. (1988). The Development of Severe Proliferative Vitreoretinopathy after Retinal Detachment Surgery. Grade B: A Determining Risk Factor. Graefes Arch. Clin. Exp. Ophthalmol..

[43] Fournier P., Aracil P., Bonnet M. (1988). Rhegmatogenous Retinal Detachment and Retinal Tear with Intravitreous Hemorrhage. J. Fr. Ophtalmol..

[44] Di Lauro S., Kadhim M.R., Charteris D.G., Pastor J.C. (2016). Classifications for Proliferative Vitreoretinopathy (PVR): An Analysis of Their Use in Publications over the Last 15 Years. J. Ophthalmol..

[45] Zhou M., Geathers J.S., Grillo S.L., Weber S., Wang W.W., Zhao Y., Sundström J., Weber S.R., Wang W., Zhao Y. (2020). Role of Epithelial-Mesenchymal Transition in Retinal Pigment Epithelium Dysfunction. Front. Cell Dev. Biol..

[46] Zandi S., Pfister I.B., Traine P.G., Tappeinerid C., Despont A., Rieben R., Skowronskaid M., Garwegid J.G. (2019). Biomarkers for PVR in Rhegmatogenous Retinal Detachment. PLoS ONE.

[47] Josifovska N., Lumi X., Szatmari-Tóth M., Kristóf E., Russell G., Nagymihály R., Anisimova N., Malyugin B., Kolko M., Ivastinović D. (2019). Clinical and Molecular Markers in Retinal Detachment-From Hyperreflective Points to Stem Cells and Inflammation. PLoS ONE.

[48] El Ghrably I. (2004). Apoptosis in Proliferative Vitreoretinopathy. Investig. Ophthalmol. Vis. Sci..

[49] Dieudonne´ S.C., La Heij E.C., Diederen R., Kessels A.G.H., Liem A.T.A., Kijlstra A., Hendrikse F. (2004). High TGF-Β2 Levels during Primary Retinal Detachment May Protect against Proliferative Vitreoretinopathy. Investig. Ophthalmol. Vis. Sci..

[50] Roldán-Pallarés M., Rollín R., Mediero A., Martínez-Montero J.C., Fernández-Cruz A., Bravo-Llata C., Fernández-Durango R. (2005). Immunoreactive ET-1 in the Vitreous Humor and Epiretinal Membranes of Patients with Proliferative Vitreoretinopathy. Mol. Vis..

[51] Shahlaee A., Yang D., Chen J., Lamy R., Stewart J.M. (2024). Vitreous Biomarkers for Proliferative Vitreoretinopathy Prognostication in Patients Undergoing Primary Retinal Detachment Repair. Transl. Vis. Sci. Technol..

[52] Wang X., Miller E.B., Goswami M., Zhang P., Ronning K.E., Karlen S.J., Zawadzki R.J., Pugh E.N., Burns M.E. (2017). Rapid Monocyte Infiltration Following Retinal Detachment Is Dependent on Non-Canonical IL6 Signaling through Gp130. J. Neuroinflamm..

[53] Chaudhary R., H Scott R.A., Wallace G., Berry M., Logan A., Blanch R.J., Blanch Neuroscience R.J., Aitken Building R. (2020). Inflammatory and Fibrogenic Factors in Proliferative Vitreoretinopathy Development. Transl. Vis. Sci. Technol..

[54] Bali E., Feron E.J., Peperkamp E., Veckeneer M., Mulder P.G., van Meurs J.C. (2010). The Effect of a Preoperative Subconjuntival Injection of Dexamethasone on Blood-Retinal Barrier Breakdown Following Scleral Buckling Retinal Detachment Surgery: A Prospective Randomized Placebo-Controlled Double Blind Clinical Trial. Graefes Arch. Clin. Exp. Ophthalmol..

[55] Tolentino F.I., Lapus J.V., Novalis G., Trempe C.L., Gutow G.S., Ahmad A. (1976). Fluorescein Angiography of Degenerative Lesions of the Peripheral Fundus and Rhegmatogenous Retinal Detachment. Int. Ophthalmol. Clin..

[56] Geller S.F., Lewis G.P., Fisher S.K. (2001). FGFR1, Signaling, and AP-1 Expression after Retinal Detachment: Reactive Müller and RPE Cells. Investig. Ophthalmol. Vis. Sci..

[57] Bastiaans J., van Meurs J.C., Mulder V.C., Nagtzaam N.M.A., Smits-te Nijenhuis M., Dufour-van den Goorbergh D.C.M., van Hagen P.M., Hooijkaas H., Dik W.A. (2014). The Role of Thrombin in Proliferative Vitreoretinopathy. Investig. Ophthalmol. Vis. Sci..

[58] Zhang J., Zhou Q., Yuan G., Dong M., Shi W. (2015). Notch Signaling Regulates M2 Type Macrophage Polarization during the Development of Proliferative Vitreoretinopathy. Cell. Immunol..

[59] Cook B.L.G.F.S.A.R. (1995). Apoptotic Photoreceptor Degeneration in Experimental Retinal Detachment. Investig. Ophthalmol. Vis. Sci..

[60] Lee J., Choi J.-H., Joo C.-K. (2013). TGF-Β1 Regulates Cell Fate during Epithelial–Mesenchymal Transition by Upregulating Survivin. Cell Death Dis..

[61] Anderson D.H., Stern W.H., Fisher S.K., Erickson P.A., Borgula G.A. (1981). The Onset of Pigment Epithelial Proliferation after Retinal Detachment. Investig. Ophthalmol. Vis. Sci..

[62] Kanda A., Noda K., Hirose I., Ishida S. (2019). TGF-β-SNAIL Axis Induces Müller Glial-Mesenchymal Transition in the Pathogenesis of Idiopathic Epiretinal Membrane. Sci. Rep..

[63] El-Asrar A.M.A., Struyf S., Van Damme J., Geboes K. (2008). Circulating Fibrocytes Contribute to the Myofibroblast Population in Proliferative Vitreoretinopathy Epiretinal Membranes. Br. J. Ophthalmol..

[64] Tamiya S., Kaplan H.J. (2016). Role of Epithelial–Mesenchymal Transition in Proliferative Vitreoretinopathy. Exp. Eye Res..

[65] Liu J., Ren L., Li S., Li W., Zheng X., Yang Y., Fu W., Yi J., Wang J., Du G. (2021). The Biology, Function, and Applications of Exosomes in Cancer. Acta Pharm. Sin. B.

[66] Kuburich N.A., den Hollander P., Pietz J.T., Mani S.A. (2022). Vimentin and Cytokeratin: Good Alone, Bad Together. Semin. Cancer Biol..

[67] Derynck R., Muthusamy B.P., Saeteurn K.Y. (2014). Signaling Pathway Cooperation in TGF-β-Induced Epithelial-Mesenchymal Transition. Curr. Opin. Cell Biol..

[68] Kim K.K., Sheppard D., Chapman H.A. (2018). TGF-Β1 Signaling and Tissue Fibrosis. Cold Spring Harb. Perspect. Biol..

[69] Nelson W.J. (2009). Remodeling Epithelial Cell Organization: Transitions between Front-Rear and Apical-Basal Polarity. Cold Spring Harb. Perspect. Biol..

[70] Debnath P., Huirem R.S., Dutta P., Palchaudhuri S. (2022). Epithelial–Mesenchymal Transition and Its Transcription Factors. Biosci. Rep..

[71] Li H., Wang H., Gu Q., Xu X. (2011). Snail Involves in the Transforming Growth Factor B1-Mediated Epithelial-Mesenchymal Transition of Retinal Pigment Epithelial Cells. PLoS ONE.

[72] Cheng H., Ho T., Chen S., Lai H., Hong K., Tsao Y. (2008). Troglitazone Suppresses Transforming Growth Factor Beta-Mediated Fibrogenesis in Retinal Pigment Epithelial Cells. Mol. Vis..

[73] Wertheimer C., Eibl-Lindner K.H., Compera D., Kueres A., Wolf A., Docheva D., Priglinger S.G., Priglinger C., Schumann R.G. (2017). A Cell Culture Technique for Human Epiretinal Membranes to Describe Cell Behavior and Membrane Contraction in Vitro. Graefes Arch. Clin. Exp. Ophthalmol..

[74] Giannandrea M., Parks W.C. (2014). Diverse Functions of Matrix Metalloproteinases during Fibrosis. Dis. Model. Mech..

[75] Chen H.-J., Ma Z.-Z. (2007). N-Cadherin Expression in a Rat Model of Retinal Detachment and Reattachment. Investig. Ophthalmol. Vis. Sci..

[76] Dongre A., Weinberg R.A. (2019). New Insights into the Mechanisms of Epithelial–Mesenchymal Transition and Implications for Cancer. Nat. Rev. Mol. Cell Biol..

[77] Yokoyama K., Kimoto K., Itoh Y., Nakatsuka K., Matsuo N., Yoshioka H., Kubota T. (2012). The PI3K/Akt Pathway Mediates the Expression of Type I Collagen Induced by TGF-Β2 in Human Retinal Pigment Epithelial Cells. Graefes Arch. Clin. Exp. Ophthalmol..

[78] Kita T., Hata Y., Arita R., Kawahara S., Miura M., Nakao S., Mochizuki Y., Enaida H., Goto Y., Shimokawa H. (2008). Role of TGF-β in Proliferative Vitreoretinal Diseases and ROCK as a Therapeutic Target. Proc. Natl. Acad. Sci. USA.

[79] Chen X., Xiao W., Liu X., Zeng M., Luo L., Wu M., Ye S., Liu Y. (2014). Blockade of Jagged/Notch Pathway Abrogates Transforming Growth Factor Β2-Induced Epithelial-Mesenchymal Transition in Human Retinal Pigment Epithelium Cells. Curr. Mol. Med..

[80] Chen X., Xiao W., Wang W., Luo L., Ye S., Liu Y. (2014). The Complex Interplay between ERK1/2, TGFβ/Smad, and Jagged/Notch Signaling Pathways in the Regulation of Epithelial-Mesenchymal Transition in Retinal Pigment Epithelium Cells. PLoS ONE.

[81] Zarubin T., Han J. (2005). Activation and Signaling of the P38 MAP Kinase Pathway. Cell Res..

[82] Eastlake K., Banerjee P.J., Angbohang A., Charteris D.G., Khaw P.T., Limb G.A. (2016). Müller Glia as an Important Source of Cytokines and Inflammatory Factors Present in the Gliotic Retina during Proliferative Vitreoretinopathy. Glia.

[83] Tanihara H., Inatani M., Honda Y. (1997). Growth Factors and Their Receptors in the Retina and Pigment Epithelium. Prog. Retin. Eye Res..

[84] Yang J.B.X. (2022). Research Progress of MicroRNA in Proliferative Vitreoretinopathy. Int. Eye Sci..

[85] Li M., Li H., Yang S., Liao X., Zhao C., Wang F. (2021). MicroRNA-29b Participates in the Epithelial-mesenchymal Transition of Retinal Pigment Epithelial Cells through P-p65. Exp. Ther. Med..

[86] Han X.-D., Jiang X.-G., Yang M., Chen W.-J., Li L.-G. (2022). MiRNA-124 Regulates Palmitic Acid-induced Epithelial-mesenchymal Transition and Cell Migration in Human Retinal Pigment Epithelial Cells by Targeting LIN7C. Exp. Ther. Med..

[87] Carpineto P., Di Filippo E.S., Aharrh Gnama A., Bondi D., Iafigliola C., Licata A.M., Fulle S. (2023). MicroRNA Expression in Subretinal Fluid in Eyes Affected by Rhegmatogenous Retinal Detachment. Int. J. Mol. Sci..

[88] Toro M.D., Reibaldi M., Avitabile T., Bucolo C., Salomone S., Rejdak R., Nowomiejska K., Tripodi S., Posarelli C., Ragusa M. (2020). MicroRNAs in the Vitreous Humor of Patients with Retinal Detachment and a Different Grading of Proliferative Vitreoretinopathy: A Pilot Study. Transl. Vis. Sci. Technol..

[89] Hiscott P., Hagan S., Heathcote L., Sheridan C.M., Groenewald C.P., Grierson I., Wong D., Paraoan L. (2002). Pathobiology of Epiretinal and Subretinal Membranes: Possible Roles for the Matricellular Proteins Thrombospondin 1 and Osteonectin (SPARC). Eye.

[90] Fisher S.K., Erickson P.A., Lewis G.P., Anderson D.H. (1991). Intraretinal Proliferation Induced by Retinal Detachment. Investig. Ophthalmol. Vis. Sci..

[91] Stern J., Temple S. (2015). Retinal Pigment Epithelial Cell Proliferation. Exp. Biol. Med..

[92] Wynn T.A. (2008). Cellular and Molecular Mechanisms of Fibrosis. J. Pathol..

[93] Amarnani D., Machuca-Parra A.I., Wong L.L., Marko C.K., Stefater J.A., Stryjewski T.P., Eliott D., Arboleda-Velasquez J.F., Kim L.A. (2017). Effect of Methotrexate on an In Vitro Patient-Derived Model of Proliferative Vitreoretinopathy. Investig. Ophthalmol. Vis. Sci..

[94] Sheridan C.M., Magee R.M., Hiscott P.S., Hagan S., Wong D.H., McGalliard J.N., Grierson I. (2002). The Role of Matricellular Proteins Thrombospondin-1 and Osteonectin during RPE Cell Migration in Proliferative Vitreoretinopathy. Curr. Eye Res..

[95] Charteris D.G., Hiscott P., Grierson I., Lightman S.L. (1992). Proliferative Vitreoretinopathy: Lymphocytes in Epiretinal Membranes. Ophthalmology.

[96] Laich Y., Wolf J., Hajdu R.I., Schlecht A., Bucher F., Pauleikhoff L., Busch M., Martin G., Faatz H., Killmer S. (2022). Single-Cell Protein and Transcriptional Characterization of Epiretinal Membranes From Patients With Proliferative Vitreoretinopathy. Investig. Ophthalmol. Vis. Sci..

[97] Morrison N., Gugerty L., Gerhart J.V., Telander D., Bravo-Nuevo A., George-Weinstein M. (2020). Myo/Nog Cells Are Present in Membranes from Patients with Proliferative Vitreoretinopathy. Investig. Ophthalmol. Vis. Sci..

[98] Charteris D.G., Hiscott P., Robey H.L., Gregor Z.J., Lightman S.L., Grierson I. (1993). Inflammatory Cells in Proliflerative Vitreoretinopathy Subretinal Membranes. Ophthalmology.

[99] Vidinova C., Voinov L., Vidinov N. (2005). Alterations in the Structure of the Epiretinal Membranes in PVR—Assumptions and Reality. Klin. Monatsblatter Augenheilkd..

[100] Abu El-Asrar A.M., Missotten L., Geboes K. (2011). Expression of Myofibroblast Activation Molecules in Proliferative Vitreoretinopathy Epiretinal Membranes. Acta Ophthalmol..

[101] Qin D., Jin X., Jiang Y. (2020). Gremlin Mediates the TGF-β-induced Induction of Profibrogenic Genes in Human Retinal Pigment Epithelial Cells. Exp. Ther. Med..

[102] Zhao F., Gandorfer A., Haritoglou C., Scheler R., Schaumberger M.M., Kampik A., Schumann R.G. (2013). Epiretinal Cell Proliferation in Macular Pucker and Vitreomacular Traction Syndrome. Retina.

[103] Fischer A.J., Zelinka C., Milani-Nejad N. (2015). Reactive Retinal Microglia, Neuronal Survival, and the Formation of Retinal Folds and Detachments. Glia.

[104] Lin M., Li Y., Li Z., Lin J., Zhou X., Liang D. (2011). Macrophages Acquire Fibroblast Characteristics in a Rat Model of Proliferative Vitreoretinopathy. Ophthalmic Res..

[105] Jones C.H., Gui W., Schumann R.G., Boneva S.K., Lange C.A.K., van Overdam K.A., Chui T.Y.P., Rosen R.B., Engelbert M., Sebag J. (2022). Hyalocytes in Proliferative Vitreo-Retinal Diseases. Expert. Rev. Ophthalmol..

[106] Lange C., Boneva S., Wieghofer P., Sebag J. (2024). Hyalocytes—Guardians of the Vitreoretinal Interface. Graefes Arch. Clin. Exp. Ophthalmol..

[107] Miller C.G., Henderson M., Mantopoulos D., Leskov I., Greco T., Schwarzbauer J.E., Prenner J.L. (2021). The Proteome of Preretinal Tissue in Proliferative Vitreoretinopathy. Ophthalmic Surg. Lasers Imaging Retin..

[108] Sramek S.J., Wallow I.H., Stevens T.S., Nork T.M. (1989). Immunostaining of Preretinal Membranes for Actin, Fibronectin, and Glial Fibrillary Acidic Protein. Ophthalmology.

[109] Ioachim E., Stefaniotou M., Gorezis S., Tsanou E., Psilas K., Agnantis N.J. (2005). Immunohistochemical Study of Extracellular Matrix Components in Epiretinal Membranes of Vitreoproliferative Retinopathy and Proliferative Diabetic Retinopathy. Eur. J. Ophthalmol..

[110] Telander D.G., Yu A.K., Forward K.I., Morales S.A., Morse L.S., Park S.S., Gordon L.K. (2016). Epithelial Membrane Protein-2 in Human Proliferative Vitreoretinopathy and Epiretinal Membranes. Investig. Ophthalmol. Vis. Sci..

[111] Cui J., Lei H., Samad A., Basavanthappa S., Maberley D., Matsubara J., Kazlauskas A. (2009). PDGF Receptors Are Activated in Human Epiretinal Membranes. Exp. Eye Res..

[112] Motulsky E., Salik D., Janssens X., Pion B., Dufrane R., Chaput F., Bolaky N., Gregoire F., Caspers L., Perret J. (2014). Aquaporin-1 Expression in Proliferative Vitreoretinopathy and in Epiretinal Membranes. Sci. World J..

[113] Yan F., Hui Y. (2012). Epidermal Growth Factor Receptor Exists in the Early Stage of Proliferative Vitreoretinopathy. Can. J. Ophthalmol..

[114] Repertinger S.K., Campagnaro E., Fuhrman J., El-Abaseri T., Yuspa S.H., Hansen L.A. (2004). EGFR Enhances Early Healing After Cutaneous Incisional Wounding. J. Investig. Dermatol..

[115] Doersch K.M., DelloStritto D.J., Newell-Rogers M.K. (2017). The Contribution of Interleukin-2 to Effective Wound Healing. Exp. Biol. Med..

[116] Harada C., Harada T., Mitamura Y., Quah H.-M.A., Ohtsuka K., Kotake S., Ohno S., Wada K., Takeuchi S., Tanaka K. (2004). Diverse NF-KappaB Expression in Epiretinal Membranes after Human Diabetic Retinopathy and Proliferative Vitreoretinopathy. Mol. Vis..

[117] Esser P., Heimann K., Bartz-Schmidt K.U., Walter P., Krott R., Weller M. (1997). Plasminogen in Proliferative Vitreoretinal Disorders. Br. J. Ophthalmol..

[118] Conforti P., Martínez Santamaría J.C., Schachtrup C. (2024). Fibrinogen: Connecting the Blood Circulatory System with CNS Scar Formation. Front. Cell. Neurosci..

[119] Laurens N., Koolwijk P., De Maat M.P.M. (2006). Fibrin Structure and Wound Healing. J. Thromb. Haemost..

[120] Sidelmann J.J., Gram J., Jespersen J., Kluft C. (2000). Fibrin Clot Formation and Lysis: Basic Mechanisms. Semin. Thromb. Hemost..

[121] Atsev S., Tomov N. (2020). Using Antifibrinolytics to Tackle Neuroinflammation. Neural Regen. Res..

[122] Yao J., Hu L.-L., Li X.-M., Shan K., Zhou R.-M., Ge H.-M., Yao M.-D., Jiang Q., Zhao C., Yan B. (2019). Comprehensive Circular RNA Profiling of Proliferative Vitreoretinopathy and Its Clinical Significance. Biomed. Pharmacother..

[123] Robbins S.G., Brem R.B., Wilson D.J., O’Rourke L.M., Robertson J.E., Westra I., Planck S.R., Rosenbaum J.T. (1994). Immunolocalization of Integrins in Proliferative Retinal Membranes. Investig. Ophthalmol. Vis. Sci..

[124] Forrester J.V., Dick A.D., McMenamin P.G., Roberts F., Pearlman E. (2016). Retina Blood Flow. The Eye.

[125] Pournaras C.J., Rungger-Brändle E., Riva C.E., Hardarson S.H., Stefansson E. (2008). Regulation of Retinal Blood Flow in Health and Disease. Prog. Retin. Eye Res..

[126] Hayreh S.S. (2011). Blood-Retinal Barrier. Acute Retinal Arterial Occlusive Disorders. Prog. Retin. Eye Res..

[127] Liu L., Liu X. (2019). Roles of Drug Transporters in Blood-Retinal Barrier. Adv. Exp. Med. Biol..

[128] Zhang K.Y., Johnson T. (2021). V The Internal Limiting Membrane: Roles in Retinal Development and Implications for Emerging Ocular Therapies. Exp. Eye Res..

[129] Lee J., Jong Seo E., Hee Yoon Y. (2022). Rhegmatogenous Retinal Detachment Induces More Severe Macular Capillary Changes than Central Serous Chorioretinopathy. Sci. Rep..

[130] Tatsumi T., Baba T., Yokouchi H., Yamamoto S. (2020). Nonperfused Peripheral Retinal Area in Eyes with Chronic Rhegmatogenous Retinal Detachment. Case Rep. Ophthalmol..

[131] Okunuki Y., Mukai R., Nakao T., Tabor S.J., Butovsky O., Dana R., Ksander B.R., Connor K.M. (2019). Retinal Microglia Initiate Neuroinflammation in Ocular Autoimmunity. Proc. Natl. Acad. Sci. USA.

[132] Chen M., Luo C., Zhao J., Devarajan G., Xu H. (2019). Immune Regulation in the Aging Retina. Prog. Retin. Eye Res..

[133] Hong S., Van Kaer L. (1999). Immune Privilege. J. Exp. Med..

[134] Streilein J.W. (2003). Ocular Immune Privilege: Therapeutic Opportunities from an Experiment of Nature. Nat. Rev. Immunol..

[135] Chen Q., Deng T., Han D. (2016). Testicular Immunoregulation and Spermatogenesis. Semin. Cell Dev. Biol..

[136] Bedogni B., Paus R. (2020). Hair(y) Matters in Melanoma Biology. Trends Mol. Med..

[137] Sonoda K.-H., Sakamoto T., Qiao H., Hisatomi T., Oshima T., Tsutsumi-Miyahara C., Exley M., Balk S.P., Taniguchi M., Ishibashi T. (2005). The Analysis of Systemic Tolerance Elicited by Antigen Inoculation into the Vitreous Cavity: Vitreous Cavity-Associated Immune Deviation. Immunology.

[138] Peterson E.J., Maltzman J.S., Koretzky G.A. (2013). T-Cell Activation and Tolerance. Clinical Immunology.

[139] Katamay R., Nussenblatt R.B. (2013). Blood–Retinal Barrier, Immune Privilege, and Autoimmunity. Retina.

[140] Maltzman J.S., Peterson E.J., Koretzky G. (2008). T-Cell Activation and Tolerance. Clinical Immunology.

[141] Zhang J., Wu G.S., Ishimoto S., Pararajasegaram G., Rao N.A. (1997). Expression of Major Histocompatibility Complex Molecules in Rodent Retina. Immunohistochemical Study. Investig. Ophthalmol. Vis. Sci..

[142] Cone R.E., Pais R. (2009). Anterior Chamber-Associated Immune Deviation (AcAID): An Acute Response to Ocular Insult Protects from Future Immune-Mediated Damage?. Ophthalmol. Eye Dis..

[143] Yin X., Zhang S., Lee J.H., Dong H., Mourgkos G., Terwilliger G., Kraus A., Geraldo L.H., Poulet M., Fischer S. (2024). Compartmentalized Ocular Lymphatic System Mediates Eye–Brain Immunity. Nature.

[144] Rusu M.C., Nicolescu M.I., Vrapciu A.D. (2022). Evidence of Lymphatics in the Rat Eye Retina. Ann. Anat..

[145] Sato T., Morishita S., Horie T., Fukumoto M., Kida T., Oku H., Nakamura K., Takai S., Jin D., Ikeda T. (2019). Involvement of Premacular Mast Cells in the Pathogenesis of Macular Diseases. PLoS ONE.

[146] Hubbard J.A., Binder D.K. (2016). Inflammation. Astrocytes and Epilepsy.

[147] Maidana D.E., Gonzalez-Buendia L., Pastor-Puente S., Naqvi A., Paschalis E., Kazlauskas A., Miller J.W., Vavvas D.G. (2023). Peripheral Monocytes and Neutrophils Promote Photoreceptor Cell Death in an Experimental Retinal Detachment Model. Cell Death Dis..

[148] Zhao Y., Weber S.R., Lease J., Russo M., Siedlecki C.A., Xu L.-C., Chen H., Wang W., Ford M., Simó R. (2018). Liquid Biopsy of Vitreous Reveals an Abundant Vesicle Population Consistent With the Size and Morphology of Exosomes. Transl. Vis. Sci. Technol..

[149] Pollreisz A., Sacu S., Eibenberger K., Funk M., Kivaranovic D., Zlabinger G.J., Georgopoulos M., Schmidt-Erfurth U. (2015). Extent of Detached Retina and Lens Status Influence Intravitreal Protein Expression in Rhegmatogenous Retinal Detachment. Investig. Ophthalmol. Vis. Sci..

[150] Dai Y., Dai C., Sun T. (2020). Inflammatory Mediators of Proliferative Vitreoretinopathy: Hypothesis and Review. Int. Ophthalmol..

[151] Kenarova B., Voinov L., Apostolov C., Vladimirova R., Misheva A. (1997). Levels of Some Cytokines in Subretinal Fluid in Proliferative Vitreoretinopathy and Rhegmatogenous Retinal Detachment. Eur. J. Ophthalmol..

[152] Kon C.H., Occleston N.L., Aylward G.W., Khaw P.T. (1999). Expression of Vitreous Cytokines in Proliferative Vitreoretinopathy: A Prospective Study. Investig. Ophthalmol. Vis. Sci..

[153] Limb G.A., Litile B.C., Meager A., Ogilvie J.A., Wolstencroft R.A., Franks W.A., Chignell A.H., Dumonde D.C. (1991). Cytokines in Proliferative Vitreoretinopathy. Eye.

[154] Balogh A., Milibák T., Szabó V., Nagy Z.Z., Kaarniranta K., Resch M.D. (2020). Immunological Biomarkers of the Vitreous Responsible for Proliferative Alteration in the Different Forms of Retinal Detachment. BMC Ophthalmol..

[155] Guo S., DiPietro L.A. (2010). Factors Affecting Wound Healing. J. Dent. Res..

[156] Opneja A., Kapoor S., Stavrou E.X. (2019). Contribution of Platelets, the Coagulation and Fibrinolytic Systems to Cutaneous Wound Healing. Thromb. Res..

[157] Wallace H.A., Basehore B.M., Zito P.M. (2023). Wound Healing Phases. StatPearls [Internet].

[158] Minutti C.M., Knipper J.A., Allen J.E., Zaiss D.M.W. (2017). Tissue-Specific Contribution of Macrophages to Wound Healing. Semin. Cell Dev. Biol..

[159] Razzell W., Evans I.R., Martin P., Wood W. (2013). Calcium Flashes Orchestrate the Wound Inflammatory Response through DUOX Activation and Hydrogen Peroxide Release. Curr. Biol..

[160] Niethammer P., Grabher C., Look A.T., Mitchison T.J. (2009). A Tissue-Scale Gradient of Hydrogen Peroxide Mediates Rapid Wound Detection in Zebrafish. Nature.

[161] Phillipson M., Kubes P. (2019). The Healing Power of Neutrophils. Trends Immunol..

[162] Wynn T.A., Vannella K.M. (2016). Macrophages in Tissue Repair, Regeneration, and Fibrosis. Immunity.

[163] Wynn T.A., Barron L. (2010). Macrophages: Master Regulators of Inflammation and Fibrosis. Semin. Liver Dis..

[164] Xue M., Jackson C.J. (2015). Extracellular Matrix Reorganization During Wound Healing and Its Impact on Abnormal Scarring. Adv. Wound Care.

[165] Alhajj M.G.A. (2022). Physiology, Granulation Tissue. StatPearls [Internet].

[166] Menko A.S., Walker J.L., Stepp M.A. (2020). Fibrosis: Shared Lessons From the Lens and Cornea. Anat. Rec..

[167] Stepp M.A., Menko A.S. (2021). Immune Responses to Injury and Their Links to Eye Disease. Transl. Res..

[168] Bonnans C., Chou J., Werb Z. (2014). Remodelling the Extracellular Matrix in Development and Disease. Nat. Rev. Mol. Cell Biol..

[169] Yue B. (2014). Biology of the Extracellular Matrix. J. Glaucoma.

[170] Rozario T., DeSimone D.W. (2010). The Extracellular Matrix in Development and Morphogenesis: A Dynamic View. Dev. Biol..

[171] Hynes R.O. (2009). The Extracellular Matrix: Not Just Pretty Fibrils. Science.

[172] Al-Ubaidi M.R., Naash M.I., Conley S.M. (2013). A Perspective on the Role of the Extracellular Matrix in Progressive Retinal Degenerative Disorders. Investig. Ophthalmol. Vis. Sci..

[173] Morikawa M., Derynck R., Miyazono K. (2016). TGF-β and the TGF-β Family: Context-Dependent Roles in Cell and Tissue Physiology. Cold Spring Harb. Perspect. Biol..

[174] Ramirez H., Patel S.B., Pastar I. (2014). The Role of TGFβ Signaling in Wound Epithelialization. Adv. Wound Care.

[175] Malmström J., Lindberg H., Lindberg C., Bratt C., Wieslander E., Delander E.-L., Särnstrand B., Burns J.S., Mose-Larsen P., Fey S. (2004). Transforming Growth Factor-Β1 Specifically Induce Proteins Involved in the Myofibroblast Contractile Apparatus. Mol. Cell. Proteom..

[176] Hata A., Chen Y.-G. (2016). TGF-β Signaling from Receptors to Smads. Cold Spring Harb. Perspect. Biol..

[177] Santibanez J.F., Kocic J. (2012). Transforming Growth Factor-β Superfamily, Implications in Development and Differentiation of Stem Cells. Biomol. Concepts.

[178] Ignotz R.A., Massagué J. (1986). Transforming Growth Factor-Beta Stimulates the Expression of Fibronectin and Collagen and Their Incorporation into the Extracellular Matrix. J. Biol. Chem..

[179] Zhu X., Kong X., Ma S., Liu R., Li X., Gao S., Ren D., Zheng Y., Tang J. (2020). TGFβ/Smad Mediated the Polyhexamethyleneguanide Areosol-Induced Irreversible Pulmonary Fibrosis in Subchronic Inhalation Exposure. Inhal. Toxicol..

[180] Alberts B.J.A.L.J. (2002). Integrins. Molecular Biology of the Cell.

[181] Ouyang X., Mehal W. Fibrogenesis. Fibrosis: Translation of Basic Research to Human Disease. https://www.sciencedirect.com/topics/medicine-and-dentistry/fibrogenesis.

[182] Walraven M., Hinz B. (2018). Therapeutic Approaches to Control Tissue Repair and Fibrosis: Extracellular Matrix as a Game Changer. Matrix Biol..

[183] Kisseleva T., Brenner D.A. (2008). Mechanisms of Fibrogenesis. Exp. Biol. Med..

[184] Carpineto P., Licata A.M., Ciancaglini M. (2023). Proliferative Vitreoretinopathy: A Reappraisal. J. Clin. Med..

[185] Friedlander M. (2007). Fibrosis and Diseases of the Eye. J. Clin. Investig..

[186] Chen H.-Y., Ho Y.-J., Chou H.-C., Liao E.-C., Tsai Y.-T., Wei Y.-S., Lin L.-H., Lin M.-W., Wang Y.-S., Ko M.-L. (2020). The Role of Transforming Growth Factor-Beta in Retinal Ganglion Cells with Hyperglycemia and Oxidative Stress. Int. J. Mol. Sci..

[187] Szczepan M., Llorián-Salvador M., Chen M., Xu H. (2022). Immune Cells in Subretinal Wound Healing and Fibrosis. Front. Cell. Neurosci..

[188] Zeng Q., Karahan E., Hondur A., Tezel T.H. (2020). Comparative Analysis of Gliosis Induced by Covering of Macular Holes with a Patch of Retinal Autograft vs Human Amniotic Membrane. Investig. Ophthalmol. Vis. Sci..

[189] Miller B. (1986). Retinal Wound Healing. Arch. Ophthalmol..

[190] Miller B., Miller H., Patterson R., Ryan S.J. (1986). Effect of the Vitreous on Retinal Wound-Healing. Graefes Arch. Clin. Exp. Ophthalmol..

[191] Hinz B. (2016). The Role of Myofibroblasts in Wound Healing. Curr. Res. Transl. Med..

[192] Purves D., Augustine G.J., Fitzpatrick D., Katz L.C., LaMantia A.-S., McNamara J.O., Williams S.M. (2001). Neuroglial cells. Neuroscience.

[193] Bianchi L., Altera A., Barone V., Bonente D., Bacci T., De Benedetto E., Bini L., Tosi G.M., Galvagni F., Bertelli E. (2022). Untangling the Extracellular Matrix of Idiopathic Epiretinal Membrane: A Path Winding among Structure, Interactomics and Translational Medicine. Cells.

[194] Hachana S., Larrivée B. (2022). TGF-β Superfamily Signaling in the Eye: Implications for Ocular Pathologies. Cells.

[195] Poniatowski Ł.A., Wojdasiewicz P., Gasik R., Szukiewicz D. (2015). Transforming Growth Factor Beta Family: Insight into the Role of Growth Factors in Regulation of Fracture Healing Biology and Potential Clinical Applications. Mediat. Inflamm..

[196] Anderson D.H., Guerin C.J., Hageman G.S., Pfeffer B.A., Flanders K.C. (1995). Distribution of Transforming Growth Factor-Beta Isoforms in the Mammalian Retina. J. Neurosci. Res..

[197] Tosi G.M., Neri G., Caldi E., Fusco F., Bacci T., Tarantello A., Nuti E., Marigliani D., Baiocchi S., Traversi C. (2018). TGF-β Concentrations and Activity Are down-Regulated in the Aqueous Humor of Patients with Neovascular Age-Related Macular Degeneration. Sci. Rep..

[198] Connor T.B., Roberts A.B., Sporn M.B., Danielpour D., Dart L.L., Michels R.G., de Bustros S., Enger C., Kato H., Lansing M. (1989). Correlation of Fibrosis and Transforming Growth Factor-Beta Type 2 Levels in the Eye. J. Clin. Investig..

[199] Thannickal V.J., Lee D.Y., White E.S., Cui Z., Larios J.M., Chacon R., Horowitz J.C., Day R.M., Thomas P.E. (2003). Myofibroblast Differentiation by Transforming Growth Factor-Beta1 Is Dependent on Cell Adhesion and Integrin Signaling via Focal Adhesion Kinase. J. Biol. Chem..

[200] de Hoz R., Rojas B., Ramírez A.I., Salazar J.J., Gallego B.I., Triviño A., Ramírez J.M. (2016). Retinal Macroglial Responses in Health and Disease. BioMed Res. Int..

[201] Burke J., Smith J. (1981). Retinal Proliferation in Response to Vitreous Hemoglobin or Iron. Investig. Ophthalmol. Vis. Sci..

[202] Fawcett J.W., Asher R.A. (1999). The Glial Scar and Central Nervous System Repair. Brain Res. Bull..

[203] Fitch M.T., Silver J. (2008). CNS Injury, Glial Scars, and Inflammation: Inhibitory Extracellular Matrices and Regeneration Failure. Exp. Neurol..

[204] Bringmann A., Iandiev I., Pannicke T., Wurm A., Hollborn M., Wiedemann P., Osborne N.N., Reichenbach A. (2009). Cellular Signaling and Factors Involved in Müller Cell Gliosis: Neuroprotective and Detrimental Effects. Prog. Retin. Eye Res..

[205] Güngör Kobat S. (2020). Importance of Müller Cells. Beyoglu Eye J..

[206] Reichenbach A., Bringmann A. (2020). Glia of the Human Retina. Glia.

[207] Buffo A., Rite I., Tripathi P., Lepier A., Colak D., Horn A.-P., Mori T., Götz M. (2008). Origin and Progeny of Reactive Gliosis: A Source of Multipotent Cells in the Injured Brain. Proc. Natl. Acad. Sci. USA.

[208] Guo L., Choi S., Bikkannavar P., Cordeiro M.F. (2022). Microglia: Key Players in Retinal Ageing and Neurodegeneration. Front. Cell. Neurosci..

[209] Wang M., Wong W.T. (2014). Microglia-Müller Cell Interactions in the Retina. Adv. Exp. Med. Biol..

[210] Fischer A.J., Reh T.A. (2003). Potential of Müller Glia to Become Neurogenic Retinal Progenitor Cells. Glia.

[211] Luna G., Lewis G.P., Banna C.D., Skalli O., Fisher S.K. (2010). Expression Profiles of Nestin and Synemin in Reactive Astrocytes and Müller Cells Following Retinal Injury: A Comparison with Glial Fibrillar Acidic Protein and Vimentin. Mol. Vis..

[212] Jing R., Wilhelmsson U., Goodwill W., Li L., Pan Y., Pekny M., Skalli O. (2007). Synemin Is Expressed in Reactive Astrocytes in Neurotrauma and Interacts Differentially with Vimentin and GFAP Intermediate Filament Networks. J. Cell Sci..

[213] Bringmann A., Pannicke T., Grosche J., Francke M., Wiedemann P., Skatchkov S., Osborne N., Reichenbach A. (2006). Müller Cells in the Healthy and Diseased Retina. Prog. Retin. Eye Res..

[214] Schachtrup C., Ryu J.K., Helmrick M.J., Vagena E., Galanakis D.K., Degen J.L., Margolis R.U., Akassoglou K. (2010). Fibrinogen Triggers Astrocyte Scar Formation by Promoting the Availability of Active TGF-β after Vascular Damage. J. Neurosci..

[215] Takeda M., Takamiya A., Jiao J., Cho K.-S., Trevino S.G., Matsuda T., Chen D.F. (2008). α-Aminoadipate Induces Progenitor Cell Properties of Müller Glia in Adult Mice. Investig. Ophthalmol. Vis. Sci..

[216] Lewis G.P., Chapin E.A., Luna G., Linberg K.A., Fisher S.K. (2010). The Fate of Müller’s Glia Following Experimental Retinal Detachment: Nuclear Migration, Cell Division, and Subretinal Glial Scar Formation. Mol. Vis..

